# Climate Change Causes Salinity To Become Determinant in Shaping the Microeukaryotic Spatial Distribution among the Lakes of the Inner Mongolia-Xinjiang Plateau

**DOI:** 10.1128/spectrum.03178-22

**Published:** 2023-06-12

**Authors:** Changqing Liu, Fan Wu, Xingyu Jiang, Yang Hu, Keqiang Shao, Xiangming Tang, Boqiang Qin, Guang Gao

**Affiliations:** a State Key Laboratory of Lake Science and Environment, Nanjing Institute of Geography and Limnology, Chinese Academy of Sciences, Nanjing, China; b University of Chinese Academy of Sciences, Beijing, China; Institut Ruder Boskovic

**Keywords:** climate change, microeukaryotic community, Inner Mongolia-Xinjiang Plateau, salinity, network stability, machine learning, network complexity

## Abstract

Climate change greatly affects lake microorganisms in arid and semiarid zones, which alters ecosystem functions and the ecological security of lakes. However, the responses of lake microorganisms, especially microeukaryotes, to climate change are poorly understood. Here, using 18S ribosomal RNA (rRNA) high-throughput sequencing, we investigated the distribution patterns of microeukaryotic communities and whether and how climate change directly or indirectly affected the microeukaryotic communities on the Inner Mongolia-Xinjiang Plateau. Our results showed that climate change, as the main driving force of lake change, drives salinity to become a determinant of the microeukaryotic community among the lakes of the Inner Mongolia-Xinjiang Plateau. Salinity shapes the diversity and trophic level of the microeukaryotic community and further affects lake carbon cycling. Co-occurrence network analysis further revealed that increasing salinity reduced the complexity but improved the stability of microeukaryotic communities and changed ecological relationships. Meanwhile, increasing salinity enhanced the importance of deterministic processes in microeukaryotic community assembly, and the dominance of stochastic processes in freshwater lakes transformed into deterministic processes in salt lakes. Furthermore, we established lake biomonitoring and climate sentinel models by integrating microeukaryotic information, which would provide substantial improvements to our predictive ability of lake responses to climate change.

**IMPORTANCE** Our findings have important implications for understanding the distribution patterns and the driving mechanisms of microeukaryotic communities among the lakes of the Inner Mongolia-Xinjiang Plateau and whether and how climate change directly or indirectly affects microeukaryotic communities. Our study also establishes the groundwork to use the lake microbiome for the assessment of aquatic ecological health and climate change, which is critical for ecosystem management and for projecting the ecological consequences of future climate warming.

## INTRODUCTION

Global climate change poses significant emerging risks to biodiversity, ecosystem function, and associated socioecological systems that are accelerating due to increasing human activity (1). Due to the sensitivity of climate change, lakes are already responding rapidly to climate, such as the warming surface water temperature and changes in evaporation and water budgets (2). Climate change endangers aquatic organisms and alters their diversity, structure, and activities (3, 4). The health and functioning of lake ecosystems rely on microbial life (5), and there is no doubt that the diversity, complexity, stability, function, and ecological relationships of microbial communities would be altered by climate change (6, 7). Although it is well documented that changes in climate affect prokaryotic diversity (3, 8), little is known about whether and how microeukaryotic communities will change under dramatic climate change (9).

As important components of aquatic organisms, microeukaryotes play a significant ecological role, such as primary producers (10, 11), bacterivores (12), parasites (13), and saprotrophs (14), in lake systems. Knowledge gaps regarding microeukaryotes hinder our predictions of climate change impacts on ecosystem functions and ecological security of lakes; microeukaryotic communities remain largely unexplored under different climate scenarios. The Inner Mongolia-Xinjiang Plateau, which is a typical arid and semiarid zone in China, has experienced unprecedented increases in land surface air temperature over the past 100 years (15, 16). Due to the larger spatial scale of the Inner Mongolia-Xinjiang Plateau, the local and climate conditions are highly diverse (17), which makes this the ideal area for exploring microeukaryotic communities under different climate conditions. A better understanding of the distribution patterns of microeukaryotic communities in this area would be beneficial for disentangling the drivers of microeukaryotic distributions under present climate change and whether and how climate change directly or indirectly affects microeukaryotic communities.

With conceptual and methodological developments, many molecular tools, especially high-throughput sequencing, have been applied to water quality monitoring and have provided a novel strategy, including environmental genomics, for lake monitoring (18, 19). High-throughput sequencing has benefited from the rapid drop in sequencing costs and time (20), which could generate massive microbial data to reflect the local and global pressures of lakes (21). However, multidimensional and noisy data sets are common in high-throughput sequencing data, which require a new modeling paradigm to convey ecological signals from background noise (19). Machine learning algorithms are adept at processing multidimensional and noisy data (22) and have attempted to prove correlations between high-throughput sequencing data and environmental stressors (23, 24). Therefore, microeukaryotic communities in lake ecosystems could be used as bioindicators to reflect ecological health and even as sentinels of present climate change.

Here, using 18S ribosomal RNA (rRNA) high-throughput sequencing, we investigated the distribution patterns of microeukaryotic communities in lakes on the Inner Mongolia-Xinjiang Plateau under different climate conditions. Our study aims to (i) understand the distribution patterns and the driving mechanisms of microeukaryotic communities, (ii) determine whether and how climate change directly or indirectly affects microeukaryotic communities, and (iii) establish lake ecological biomonitoring and climate sentinel models by integrating microeukaryotic information.

## RESULTS

### High diversity of environmental variables.

The main physicochemical and climatic parameters characterizing the lakes on the Inner Mongolia-Xinjiang Plateau are shown in Fig. S1, Fig. S2, and Table S2 in the supplemental material. All environmental variables were significantly different between the lakes (*P* < 0.001), which provided a high diversity of distinct niches and adaptations for survival. High total nitrogen (TN) concentrations (>4 mg · L^−1^) were observed in Lake Chagannur, Lake Daihai, Lake Dalinor, and Lake Ganggengnor, and high total phosphorus (TP) concentrations (>2 mg · L^−1^) were also observed in Lake Dalinor. Most monthly self-calibrating Palmer drought severity indices (MPDSIs) and annual self-calibrating Palmer drought severity indices (APDSIs) were far higher than 2 or lower than −2, which indicated extreme humid or drought conditions. Due to the extreme conditions, significant changes in lake area were observed in the sampled lakes (*P* < 0.01), except Lake Durenor and Lake Bosten. After 1960, Lake Chagannur, Lake Daihai, Lake Dalinor, and Lake Xiangsi showed a significant decrease in water area, whereas the lake areas of Lake Ganggengnor and Lake Sayram significantly expanded (Fig. S3).

### Spatial distribution of the microeukaryotic communities.

**FIG 1:**
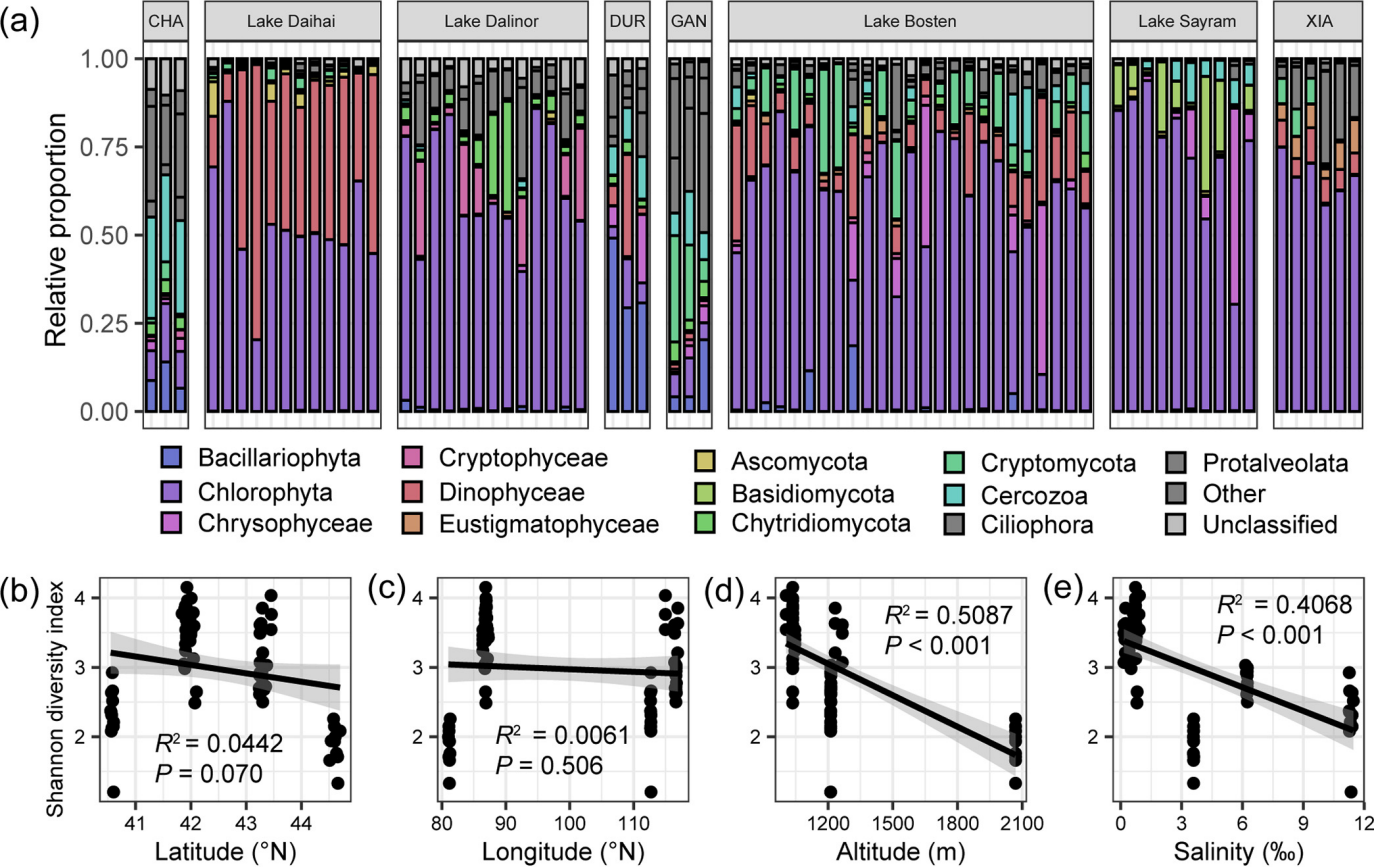
Diversity of the microeukaryotic communities among the lakes of the Inner Mongolia-Xinjiang Plateau. (a) Spatial distribution of the microeukaryotic communities. CHA, DUR, GAN, and XIA represent Lake Chagannur, Lake Durenor, Lake Ganggengnor, and Lake Xiangsi, respectively. (b to e) Relationships between Shannon diversity index and latitude (b), longitude (c), altitude (d), and salinity (e).

High-throughput sequencing yielded 2,294,101 high-quality sequences with 1,188 zero-radius operational units (ZOTUs) for the 18S rRNA V4 region (Table S3). Most sequences of 18S rRNA were mainly assigned to algae (74.23%), especially Chlorophyta (52.91%), which dominated microeukaryotic communities among most lakes except Lake Chagannur, Lake Durenor, and Lake Ganggengnor (Fig. 1).

After resampling, the ZOTU richness ranged from 60 (DAI04) to 356 (CHA02), with a mean of 164.09 (Fig. S4a). The Shannon diversity index had an average of 2.976 but varied between 1.203 (DAI04) and 4.153 (BOS20) (Fig. S4b). Significant differences in ZOTU richness and Shannon diversity were both observed between the lakes (*P* < 0.001). To discern potential spatial patterns of microeukaryotic diversity, we used linear models to estimate the relationships between geographic parameters and the Shannon index. The ZOTU richness and Shannon index decreased significantly with altitude (*P* < 0.001), although they did not show a significant latitudinal or longitudinal pattern (*P* > 0.001) (Fig. 1b to d and Fig. S5a to c).

**FIG 2:**
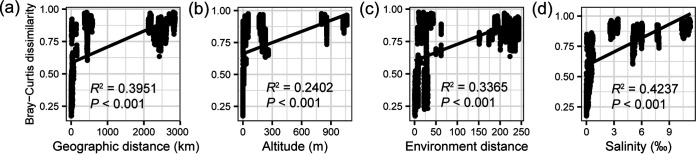
(a to d) Relationships of microeukaryotic communities (based on Bray-Curtis distances) with geographic distance (a), altitude (b), environmental distance (based on Euclidean distance) (c), and salinity (d). *R*^2^ represents the communities variations explained by geographic distance, altitude, environmental distance, and salinity, respectively.

Based on the Bray-Curtis distance, the permutational multivariate analysis of variance (PERMANOVA) results also showed that lake differences explained 84.07% of the microeukaryotic community variance (*P* < 0.001). The geographic pattern, including geographic distance (*R*^2^ = 0.3954, *P* < 0.0001) and altitude (*R*^2^ = 0.2405, *P* < 0.0001), showed a significant positive relationship with microeukaryotic Bray-Curtis dissimilarity (Fig. 2a and b). A significant dissimilarity increase was also detected with increasing environmental distance (*R*^2^ = 0.3367, *P* < 0.0001), indicating that those environmental variables may play an important role in shaping microbial spatiotemporal distributions (Fig. 2c).

### Driving factors of microeukaryotic spatial distribution.

Using stepwise multiple-regression model analysis, we found that the environmental variables were able to explain most variation in ZOTU richness (91.17%) and the Shannon index (72.24%). The physicochemical (salinity, water temperature [WT], and TP) and climatic variables (mean monthly precipitation [MMP], mean annual precipitation [MAP], and mean annual temperature [MAT]) significantly affected the ZOTU richness or Shannon index (*P* < 0.001) (Table S4). However, salinity was consistently the determining factor both for the ZOTU richness and Shannon index, whose increase would significantly reduce microeukaryotic diversity (*P* < 0.001) (Fig. 2d and Fig. S5d).

**FIG 3:**
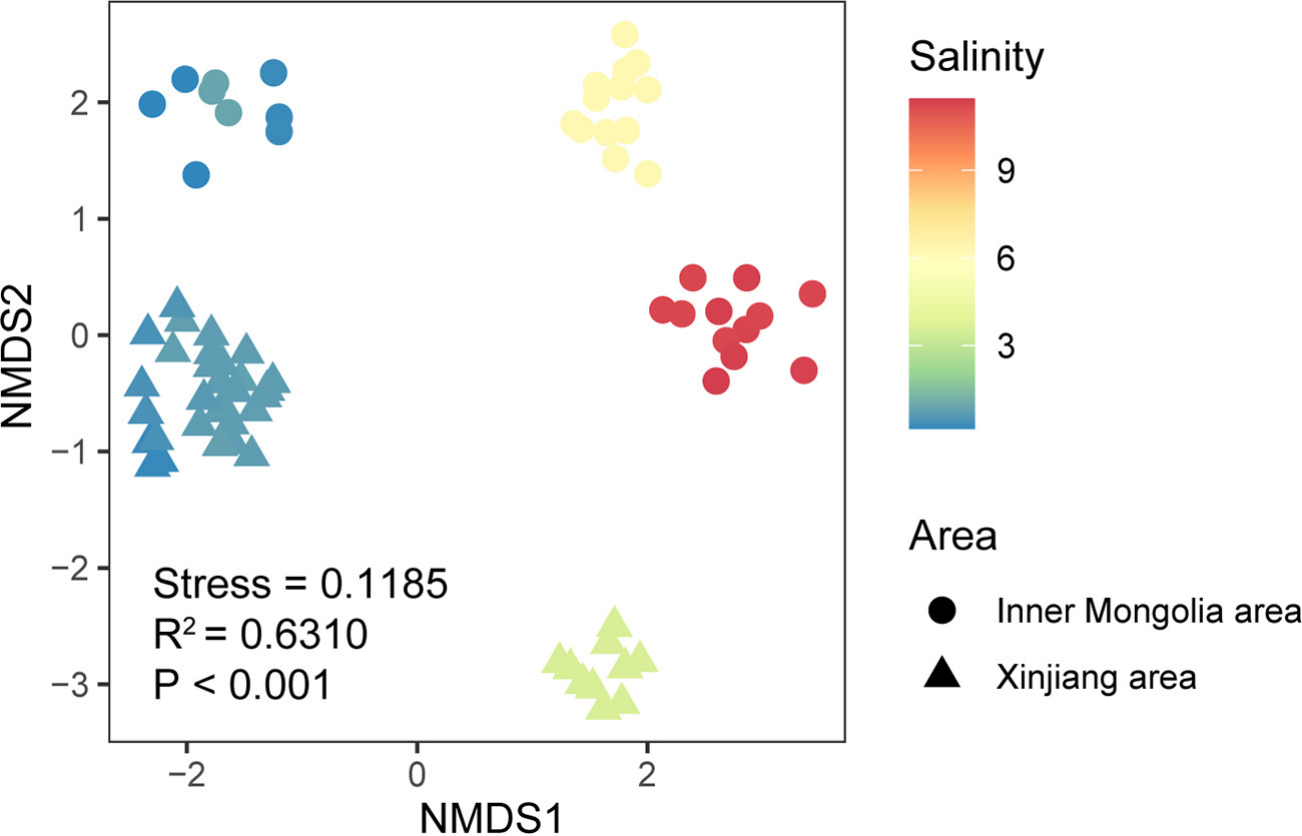
NMDS analysis of the microeukaryotic communities based on Bray-Curtis dissimilarity. The shape of the symbol shows the sampling area, and the color of the symbol indicates the salinity gradient. A significant effect of salinity on microeukaryotic communities was detected by PERMANOVA.

The NMDS ordination showed that the microeukaryotic community clustered based on salinity (Fig. 3), including salt lakes (Lake Daihai; salinity, ~11.3%), subsalt lakes (Lake Dalinor; salinity, ~6.2%), brackish lakes (Lake Sayram; salinity, ~3.6%,) and freshwater lakes (Lake Bosten, Lake Chagannur, Lake Durenor, Lake Ganggengnor, and Lake Xiangsi; salinity, <1.0%), which explained 63.10% of the microeukaryotic community variance based on the PERMANOVA results (*P* < 0.001). The microeukaryotic Bray-Curtis dissimilarity significantly increased with increasing salinity difference (*R*^2^ = 0.4239, *P* < 0.0001) (Fig. 2d).

To quantify the influence of physicochemical, climatic, and geographic parameters on the spatial distribution of the microeukaryotic community, variation partitioning analysis (VPA) was introduced in our research (Fig. S6). The results indicated that these three types of factors could explain 33.82, 31.77, and 32.44% of the observed variation (including alone and combined explanation), respectively, leaving 44.32% of the variation unexplained. Physicochemical factors alone explained 7.37% of the variation, which was higher than climatic (5.66%) and geographic parameters (5.22%). Canonical correlation analysis (CCA) was used to further explore the independent effect of the environmental factors on the microeukaryotic community (Table S5), indicating that salinity was the most important factor that determined the microeukaryotic spatial distribution. The random forest (RF) model was also used to interpret and quantify the relative importance of environmental variables to the microeukaryotic community, which revealed that salinity was the most important variable covarying with the microeukaryotic community (Fig. S7).

### Differential response of trophic and taxonomic groups to salinity stress.

**FIG 4:**
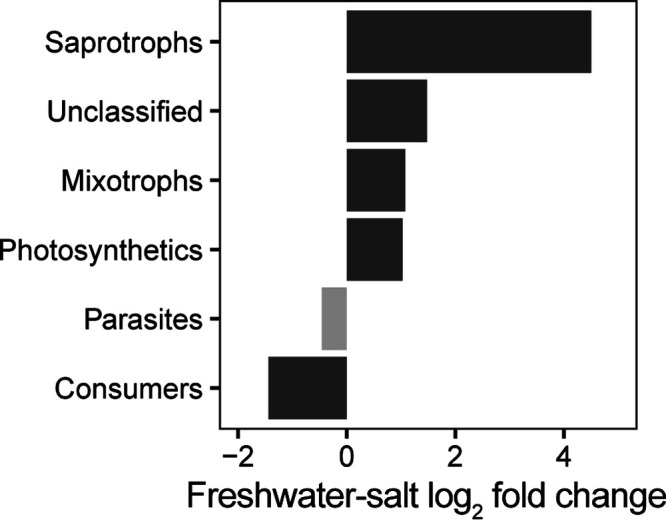
Magnitude of change in trophic groups between the freshwater and salt lakes. Dark and gray bars represent groups for which the change was found significant (*P* < 0.05) and not significant (*P* > 0.05).

Based on the results of differential abundance analysis, the significant reduction was only observed in consumers (*P* < 0.05) (Fig. 4). However, saprotrophs, the other heterotrophs, significantly increased in salt lakes (*P* < 0.05), whose increases were greater than those of other trophic groups. As the primary producer of ecosystems, the relative abundance of both mixotrophs and photosynthetic organisms significantly increased in salt lakes (*P* < 0.05).

To identify which taxonomic groups were involved in salinity stress, DESeq2 analysis was also conducted for 50 microeukaryotic classes. Most classes (41 of 50) significantly changed in the salt lakes, including 23 classes that increased and 18 classes that decreased (Fig. S8). The relative abundance of most classes belonging to fungi significantly increased in the salt lakes (*P* < 0.05), especially Tremellomycetes and Cystobasidiomycetes. Conversely, most classes belonging to consumers significantly decreased in the salt lakes, especially Aconoidasida and Postciliodesmatophora (*P* < 0.05). The tolerant groups were composed mainly of algae (*P* > 0.05), such as Chlorophyceae and Bacillariophyceae, although few classes of algae would significantly change in regard to salinity.

### Network complexity and stability of the microeukaryotic community.

**FIG 5:**
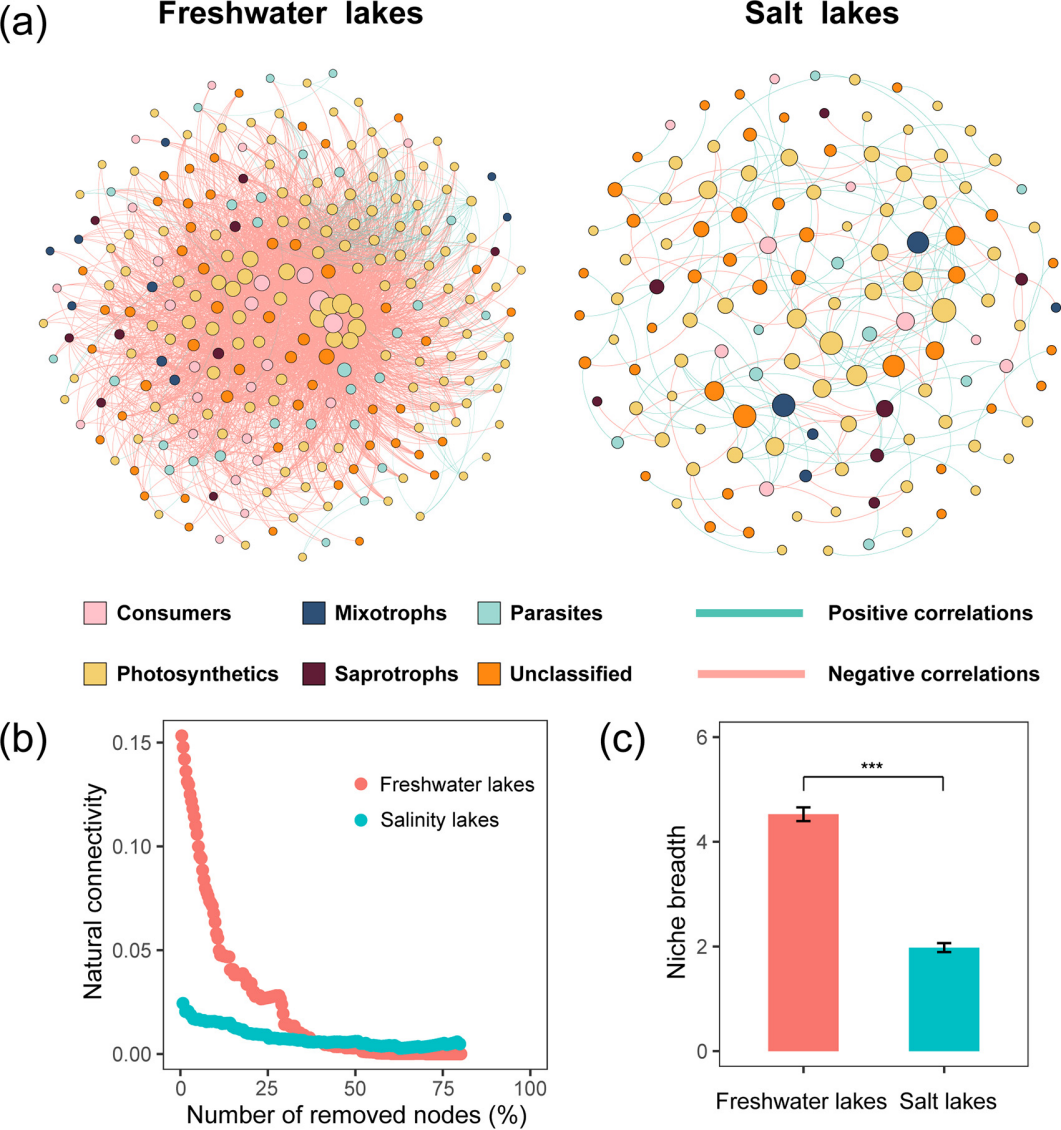
MENs and niche breadth of the microeukaryotic community in freshwater and salt lakes. (a) Network revealing the trophic associations among microeukaryotes at the ZOTU level. The size of the circles shows the degree of the node, and the color of the circles shows the trophic type of the node. The green lines show positive correlations, and the red lines show negative correlations. (b) Natural connectivity of microeukaryotic communities in freshwater and salt lakes. (c) Habitat niche breadth for all taxa in the freshwater and salt lakes. (***, significant difference at the *P* < 0.001 level using the Wilcoxon rank sum test).

The degrees of freshwater (power law, *R*^2^ = 0.637) and salt networks (power law, *R*^2^ = 0.803) followed a scale-free distribution, suggesting that both network structures were nonrandom. The modularity of the networks was significantly higher than that of their respective random networks, suggesting that both networks had modular structures (Table S6 and Fig. 5a).

To determine how salinity affected microeukaryotic network complexity, the topological parameters between freshwater and salt networks were compared (Table S6). The network size (total nodes) was higher under freshwater lakes than under salt lakes, as was the link network connectivity (total links), average connectivity (average links per node), geodesic efficiency, and connectedness. However, the modularity, average clustering coefficient, average path distance, harmonic geodesic distance, and transitivity were higher in the salt lakes. Moreover, the dominance of the interspecies interactions turned from negative in freshwater lakes to positive in salt lakes. These changes in topological parameters and interspecific interactions indicated the lower complexity and negative interactions of salt networks.

To determine the stability of the microeukaryotic networks between the freshwater and salt lakes, we simulated species extinction and calculated robustness (resistance to node loss) and carried out natural connectivity analysis. Compared with the salt lakes, the natural connectivity values decreased sharply in freshwater lakes, although they were higher at first (Fig. 5b).

Altered network complexity and stability were associated with changes in the role of individual members within the network. Based on the mean of nestedness (Zi) and among-module connectivity (Pi) of each node, 14 module hubs in the freshwater network and 3 module hubs and 3 connectors in the salt network were detected (Fig. S9), all of which were regarded as keystone nodes that play key roles in shaping network structure. The keystone nodes were affiliated with photosynthetic organisms and consumers in the freshwater network; however, the keystone nodes in the salt network were affiliated with photosynthetic organisms, saprotrophs, and unclassified organisms. Importantly, keystone nodes were also not preserved between freshwater and salt lakes, and both networks did not share the same keystone nodes.

Furthermore, the node degree proportions of consumers, parasites, and photosynthetic organisms were lower in the salt lakes, especially the consumers, which decreased by over half (Fig. S10). Instead, the mixotrophs, saprotrophs, and unclassified organisms were higher in the salt lakes. The interspecies interactions between the mixotrophs and other trophic groups increased with increasing salinity; however, the interactions between the consumers and other trophic groups (except mixotrophs) were weak (Fig. S11). The main primary producer, photosynthetic organisms, could increase the interactions with mixotrophs and saprotrophs but decreased the interactions with consumers and parasites.

### Assembly processes of the microeukaryotic community.

The relationships between the β-nearest taxon index (βNTI) and major environmental parameters were used to infer changes in the relative influences of environmental factors on deterministic and stochastic assembly processes. The partial Mantel test showed that physicochemical parameters (*P* < 0.05) and geographic pattern (*P* < 0.01) significantly influenced βNTI when holding other effects (Table S7).

Furthermore, we quantified the assembly processes of microeukaryotic communities by combining βNTI and Bray-Curtis-based Raup-Crick (RC_Bray_). The stochastic processes (55.51%) could dominate the freshwater communities, while the dominance could transform into deterministic processes (63.19%) in regard to salinity (Fig. S12). Heterogeneous selection contributed the largest fraction to the microeukaryotic community assembly of both freshwater (42.82%) and salt (63.03%) lakes, with higher contributions to salt communities. The influence of dispersal limitation (freshwater lakes, 27.31%; salt lakes, 12.94%) and undominated (24.23%, 12.11%) could decrease when salinity increased; conversely, the importance of homogenizing dispersal increased (3.97%, 11.76%). With the shift in community assembly, the niche breadths of microeukaryotic communities also significantly decreased when salinity increased (Wilcoxon rank sum test, *P* < 0.001) (Fig. 5c).

### The direct and indirect relationships between environmental factors and microeukaryotic communities.

**FIG 6:**
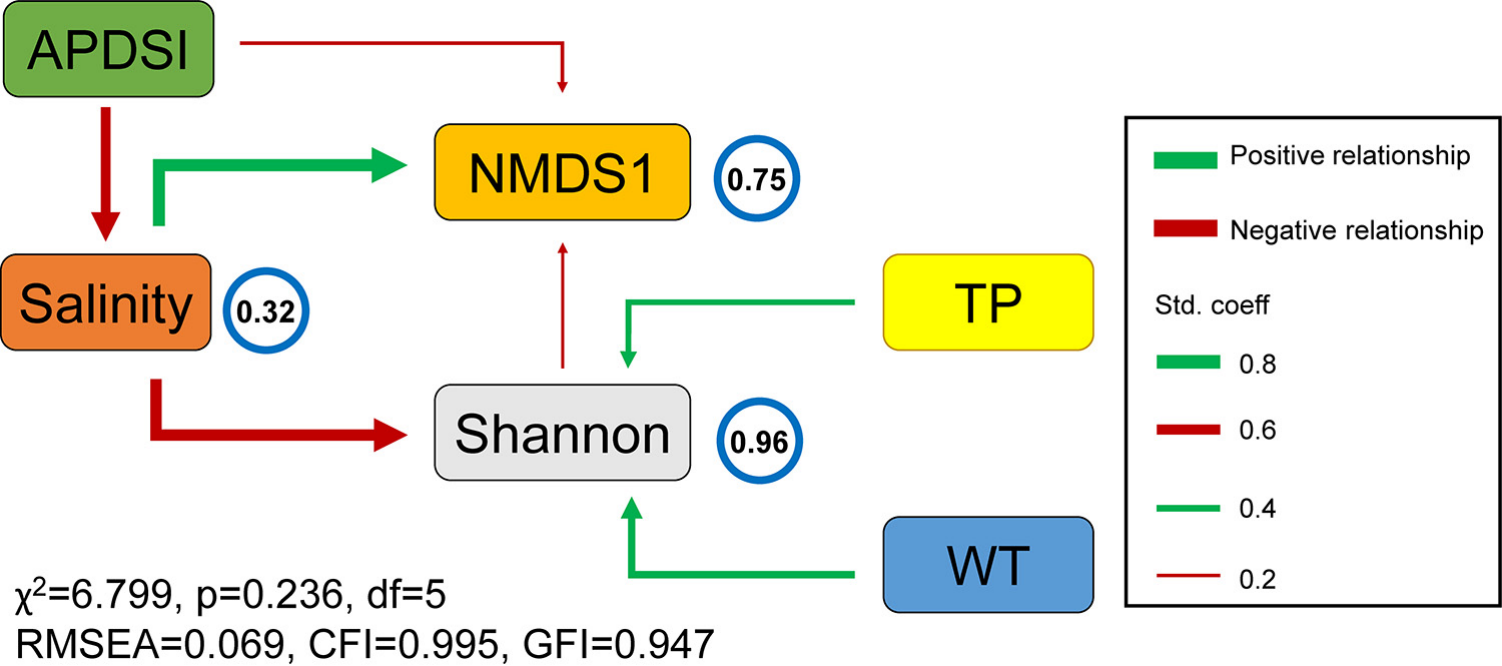
SEM describing the direct and indirect relationships among the physicochemical variables, climatic factors, and microeukaryotic community. The arrow width is proportional to the strength of the path coefficients. The numbers in the blue circle presented above every response variable in the model denote the proportion of variance.

Due to the intimate connection between climatic and physicochemical factors, we conducted structural equation modeling (SEM) to test possible direct and indirect relationships between environmental factors and microeukaryotic communities (Fig. 6). Consistent with the previous results, salinity had the strongest direct effects on the Shannon index (standardized path coefficient; β, −0.66; *P* < 0.0001) and nonmetric multidimensional scaling (NMDS1) (β = 0.70, *P* < 0.001), and the Shannon index also directly affected NMDS1 (β, −0.18; *P* < 0.001). Although the APDSI had a significant effect on NMDS1 (β, −0.21; *P* < 0.001), it was also strongly correlated with salinity (β, −0.57; *P* < 0.001) and indirectly affected NMDS1. In addition, the other physicochemical factors, WT (β = 0.43, *P* < 0.001) and TP (β = 0.33, *P* < 0.001), had positive impacts on the Shannon index.

### Microeukaryotes serve as biomarkers to indicate lake characteristics and climate change.

**FIG 7:**
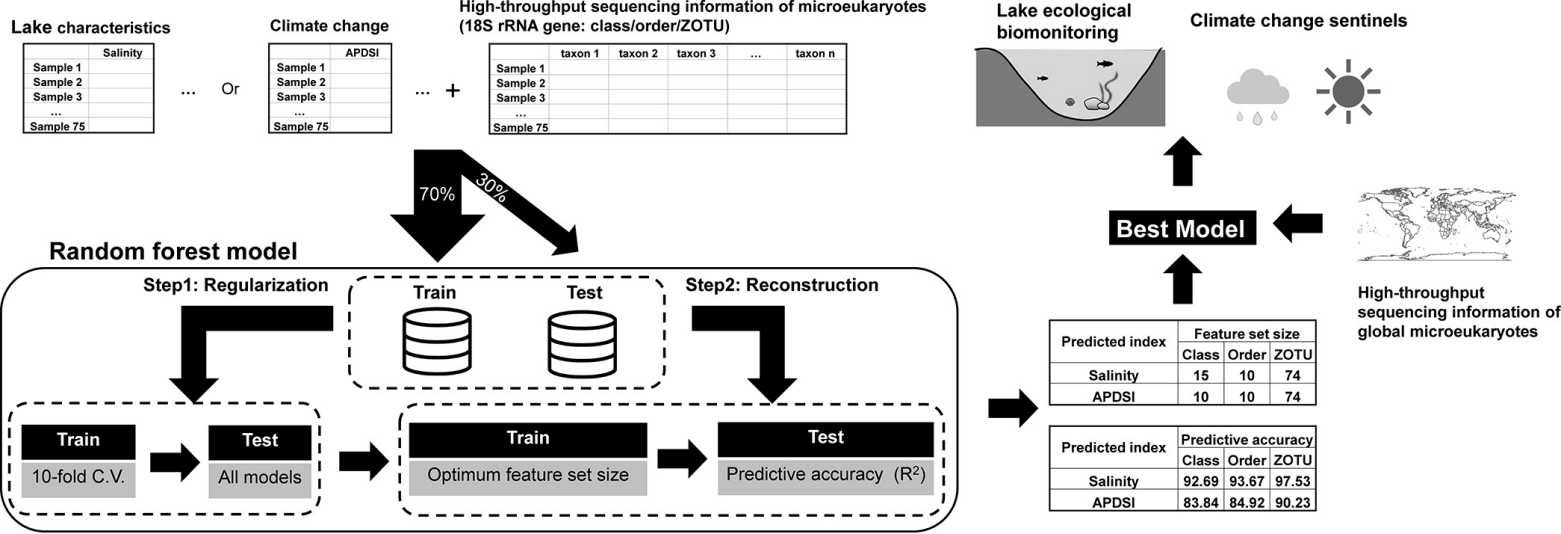
Schematic outlining the machine learning workflow used to create RF models for lake characteristics and climate change using different microeukaryotic feature sets.

To explore whether microeukaryotes can be used as biomarkers to indicate lake characteristics and climate change, we constructed RF models for salinity and APDSI at different taxonomic resolutions (Fig. 7 and Fig. S13 and 14). All models performed well at predicting with predictive accuracy above 80%, especially for salinity (above 90%). Based on 10-fold cross-validation, we identified the optimum feature set size to reconstruct new models for different taxonomic resolutions, which was slightly higher than that of the model constructed using all feature sets. Models trained at lower taxonomic resolution were also significantly less accurate and showed a larger increase in prediction accuracy for salinity (4.84% increase) and APDSI (6.39% increase) between class and ZOTU rank.

Based on the results described above, we used microeukaryotic data resolved at the ZOTU rank with 74 feature set sizes to construct the best model to predict salinity (97.53%) and APDSI (90.23%) (Fig. S13 and 14). Both models had excellent prediction accuracy, which provided insights into lake ecological biomonitoring and climate change sentinels around the world by combining the public data of global microeukaryotes.

## DISCUSSION

### Salinity is a determinant in shaping the microeukaryotic spatial distribution under different climate conditions.

The Inner Mongolia-Xinjiang Plateau, where lakes are widely distributed, is a typical arid and semiarid zone area in China. Due to the large spatial scale, the Inner Mongolia-Xinjiang Plateau possesses complex climatic and geographic conditions (see Tables S1 and S2 and Fig. S1 to S3 in the supplemental material), which proved the high diversity of lake habitats. In this study, the main physicochemical and climatic parameters of lakes on the Inner Mongolia-Xinjiang Plateau were significantly different (Table S2), which provided a high diversity of distinct niches and adaptations for survival. Similar to bacterial and fungal communities (25, 26), the microeukaryotic community distribution was strongly affected by geographic location and showed significant distance-decay relationships in the lakes of the Inner Mongolia-Xinjiang Plateau (Fig. 2). The geographic distribution of the microeukaryotic community could be driven by multiple factors, including dispersal limitation at a large spatial scale (27), local conditions of lakes (pH, salinity, nutrition, etc.) (26, 28, 29), and stochastic processes of community assembly (30).

In our study, salinity was determined to be a factor for microeukaryotic diversity, which increased the loss of diversity in the microeukaryotic community (Table S5 and Fig. 1e). Generally, salinity was regarded as the main environmental stress of the microbial community, which could reduce the niches of the microeukaryotic community and further reduce diversity (Fig. 1e and Fig. 5c). Specifically, due to the rise in extracellular osmolarity (31–33) and the generation of reactive oxygen species (34) with increasing salinity, most microeukaryotes are likely to die or become less active, leading to a decrease in community diversity.

Based on the results of the VPA and RF models (Fig. S6 and S7), salinity also became the key determinant for microeukaryotic community structure on the Inner Mongolia-Xinjiang Plateau. As an environmental stress, different salinity ranges are usually inhabited by distinct microbial communities due to the specific tolerance or requirement of microorganisms (31). Compared with salt lakes, freshwater lakes possess higher primary productivity (35), which provides a wider niche breadth for consumers. After salinity increased, the relative abundance of consumers decreased due to niche shrinkage (Fig. 5c). However, the saprotrophic fungi were more adapted and favored salinity stress compared with other microorganisms (36), and their relative abundance significantly increased in the salt lakes (Fig. 4).

Climate change is one of the most severe threats to global lake ecosystems (2), generating complex responses that vary in geographic distribution, magnitude, and local conditions of lakes around the world (37). The Inner Mongolia-Xinjiang Plateau, a region sensitive and vulnerable to global climate change, has aquatic ecosystems that have been acutely disturbed by climate change. One of the most visible examples is the extreme humid or drought conditions since the hydrological imbalance is induced by changing temperature and precipitation (Fig. S2). The SEM results further confirmed that the drought condition (APDSI) not only had a direct effect on the microeukaryotic communities but also an indirect effect by the decisive role of lake salinity (Fig. 6). Thus, climate change could lead to remarkable lake shrinkage (Fig. S3) and cause salinity changes, which drove salinity to become a determinant of the microeukaryotic community among the lakes of the Inner Mongolia-Xinjiang Plateau. The rate and magnitude of climate change in recent years and in the projected future are unprecedented (2), which severely exacerbates changes in lake salinity and increases the salinity stress of the microeukaryotic community.

### Higher salinity reduces the complexity but improves the stability of microeukaryotic communities.

The complicated ecological relationships in lakes could be represented as networks, which are fundamental for characterizing the species interactions and dynamics of ecosystems (38). Therefore, a network of salt and freshwater lakes was constructed to explore whether ecological relationships change in microeukaryotic communities under different salinity ranges. Both networks are scale free and modular (Table S6), which implies strong interspecies interactions between microeukaryotic communities and may have profound implications for microbially mediated ecosystem functions and stability (39, 40).

Higher salinity reduced the complexity of the microeukaryotic network, which had lower size, connectivity, and average connectivity (Table S6). Contrary to the network complexity reduction, a rapid increase in positive interactions was found in salt lakes (Table S6). This dominance of the positive interactions could be explained by two nonmutually exclusive mechanisms, species interactions and environmental filtering (41, 42). Based on the stress gradient hypothesis (43), the taxa that tend to be involved in antagonistic interspecific interactions are replaced by slow-growing, stress-tolerant species and mutualistic species when salinity stress increases. Usually, the interspecific interactions between consumers and other trophic groups tend to be antagonistic, such as predator-prey relationships among consumers and photosynthetic organisms. Weaker interspecific interactions were observed in salt lakes, which proved the replacement of mutualism under salinity stress (Fig. S11). Environmental filtering (salinity stress) reduces the available habitats for microeukaryotes, which is also supported by the niche breadth (Fig. 5c). Usually, the positive correlations among taxa possessed a similarity of niches (44, 45), which would be more adapted to shrunken niches. Therefore, the antagonistic interaction loss and environmental filtering increase caused by salinity stress would drive the transformation of interspecies interactions among the microeukaryotic community.

Environmental stress not only has the potential to reorganize the interactions between coexisting microbial taxa (complexity) of networks but also the properties (stability) of these networks in response to disturbances (39, 46). The complexity and stability of microbial networks have been widely explored in recent years, but the relationships between network complexity and stability remain controversial (39, 47). The network with lower connectivity and centrality but higher modularity (Table S6) indicates the higher stability of microeukaryotic communities in salt lakes (48). In addition, the higher robustness of salt lakes (Fig. 5b) further confirmed that the stability of microeukaryotic communities improved with increasing salinity (49). Our results provide explicit evidence that network stability decreased with network complexity, which was consistent with the theoretical analysis showing that higher complexity destabilizes ecological systems (50).

### Salinity shapes trophic levels and microbial food webs.

Inland waters, especially lakes, play an important role in global carbon cycling (51), whose flux controls the composition of trophic levels and experiences significant disruptions from climate change. In our study, changes along salinity gradients drove not only community diversity but also the trophic interactions of microeukaryotes (Fig. S10 and 11), which indicated a change in carbon cycling in the lake. Both the relative abundance and functional importance (network degree) of consumers were reduced in the salt lakes (Fig. 4 and Fig. S10). These consumers could be consumed by larger zooplankton, which allows an energy transfer from the microbial food web into the classic food chain (52). Therefore, the significant loss of consumers may indicate a lower efficiency in passing primary production to higher trophic levels when salinity increased.

According to the microbial carbon pump hypothesis (53), the loss of consumers also means that a significant fraction of primary production may transfer to microorganisms, such as bacterial metabolism (53), viral lysis (54), fungal parasitism (55), and saprophytism (13). The significant increase in saprotrophs in the salt lakes (Fig. 4 and Fig. S10) would further support this change in carbon cycling. Considering the inconsistent salinization trend of lakes on the Inner Mongolia-Xinjiang Plateau, the estimation of the carbon budget and the achievement of carbon neutrality would be more challenging under the unprecedented rate of climate change.

### Increasing salinity improves the importance of deterministic processes in microeukaryotic community assembly.

The balance between deterministic and stochastic processes influences the assembly of species in communities (56), and the central challenge in biogeography becomes how to quantify the contributions of deterministic and stochastic processes to microbial community assembly (57). In lakes on the Inner Mongolia-Xinjiang Plateau, geographic patterns and physicochemical parameters significantly influenced the assembly of the microeukaryotic community (Table S7), and both were generally considered the key factor for the balance between deterministic and stochastic processes (58). Usually, the importance of deterministic processes decreases with increasing scale since stronger environmental gradients would lead to deterministic structuring (59, 60). With the increase of climate change and human activities (61), lakes will experience dramatic changes in the future, which will favor the dominance of deterministic processes.

Environmental filtering can influence assembly processes of the microbial community (62). On the Inner Mongolia-Xinjiang Plateau, increasing salinity would improve the importance of deterministic processes in microeukaryotic community assembly, and the dominance of stochastic processes in freshwater lakes would transform into deterministic processes in salt lakes (Fig. S12). As environmental filtering, salinity stress reduces the available habitats (niche breadth) for microeukaryotes (Fig. 5c), which enhances the importance of deterministic processes in microeukaryotic community assembly (63). Usually, environmental conditions change across space or time, and high variation in community structure, could exist, which is referred to as heterogeneous selection (57). Larger geographic scales, more dramatic climate change, and intensive human activities result in heterogeneous habitats on the Inner Mongolia-Xinjiang Plateau (61). As expected, heterogeneous selection was found to be the main process driving the assembly of both freshwater and salt lakes. Due to the high diversity of ion types (64) and salinization processes (65), the habitats of salt lakes are more heterogeneous than those of freshwater lakes. Therefore, heterogeneous selection was more important in salt lakes.

### Microeukaryotic information provided insights into lake ecological biomonitoring and climate change scenarios.

Microbial communities can be sensitive indicators of environmental change and dysbiosis and have gradually been integrated into routine aquatic biomonitoring (18). In our study, the microeukaryotic community in the lakes of the Inner Mongolia-Xinjiang Plateau was closely related to lake characteristics. Using machine learning algorithms, we successfully constructed a prediction model based on the microeukaryotic information derived from 18S rRNA gene sequencing, which exhibited excellent prediction accuracy for lake salinity (Fig. 7). The size-dispersal hypothesis argues that smaller organisms (bacteria) are more environmentally filtered than larger organisms (microeukaryotes) (66). Therefore, this hypersensitivity leads to absence of almost bacteria in extreme environments (such as hypersaline or extreme pH conditions) (67) and further affects accuracy of the prediction model based on the bacterial information. Compared with the bacterial community (23), microeukaryotes perform better in aquatic ecological prediction and are more adapted to routine biomonitoring.

Expanding ecological assessment by integrating microeukaryotes into routine biomonitoring would improve our global understanding of lake responses to a changing climate. As the lowest points in the surrounding landscape, lakes could comprise a large, geographically distributed network of sensors that provide response information of terrestrial and aquatic ecosystems to climate change (37, 68). Climate-driven changes in the microeukaryotic community were remarkable on the Inner Mongolia-Xinjiang Plateau (Fig. 6), which implied that microeukaryotic information could be used as climate change sentinels. Based on the RF model, we successfully established the climate sentinel model by integrating lake microeukaryotes (Fig. 7), which is critical for ecosystem management and for projecting the ecological consequences of future climate warming on the Inner Mongolia-Xinjiang Plateau. Benefiting from the widespread application and data accumulation of high-throughput sequencing (26, 69), we could even establish a global climate model by integrating global microeukaryotic and prokaryotic information from microbial genomic databases. However, considering microbial information bias caused by sampling and sequencing methods, a consistent and generalized approach should be established in global work.

### Conclusion.

The rate and magnitude of climate change in recent years and in the projected future are unprecedented, which severely threatens global lake ecosystems (2), especially in climate-sensitive areas (arid and semiarid zones) (15, 16). Our findings have important implications for understanding the distribution patterns and the driving mechanisms of microeukaryotic communities among the lakes of the Inner Mongolia-Xinjiang Plateau and whether and how climate change directly or indirectly affects microeukaryotic communities. On the Inner Mongolia-Xinjiang Plateau, climate change drove salinity to become a determinant of the microeukaryotic community among the lakes. The results showed a significant salinity decay pattern in the microeukaryotic community, which could be clustered based on salinity gradients. As the deterministic filtering factor, increasing salinity resulted in divergent succession with reduced stochasticity and caused the domination of deterministic processes in salt lakes. Furthermore, increasing salinity reduced the complexity but improved the stability of the microeukaryotic network; however, this relationship between complexity and stability was contrary to the bacterial network. Our results also found a loss of consumers in salt lakes, which implied a decrease in energy transfer to higher trophic levels and a change in lake carbon cycling.

Benefiting from the microeukaryotic community having a strong association with lake characteristics and climate change, we established lake biomonitoring and climate sentinel models by integrating microeukaryotic information on the Inner Mongolia-Xinjiang Plateau. Benefiting from the widespread application and data accumulation of high-throughput sequencing, we could even establish a microeukaryotic indicator of climate change at the global scale. In addition, revealing the natural processes of past climate change is critical for ecosystem management and for projecting the ecological consequences of future climate warming; however, the lack of long-term lake and climate data sets is common around the world. Considering the good DNA preservation in sediment and climate prediction of microeukaryotes, it is now possible to reconstruct past microeukaryotic assemblages and infer historic climate change using the RF model.

## MATERIALS AND METHODS

### Sampling and environmental information.

**FIG 8:**
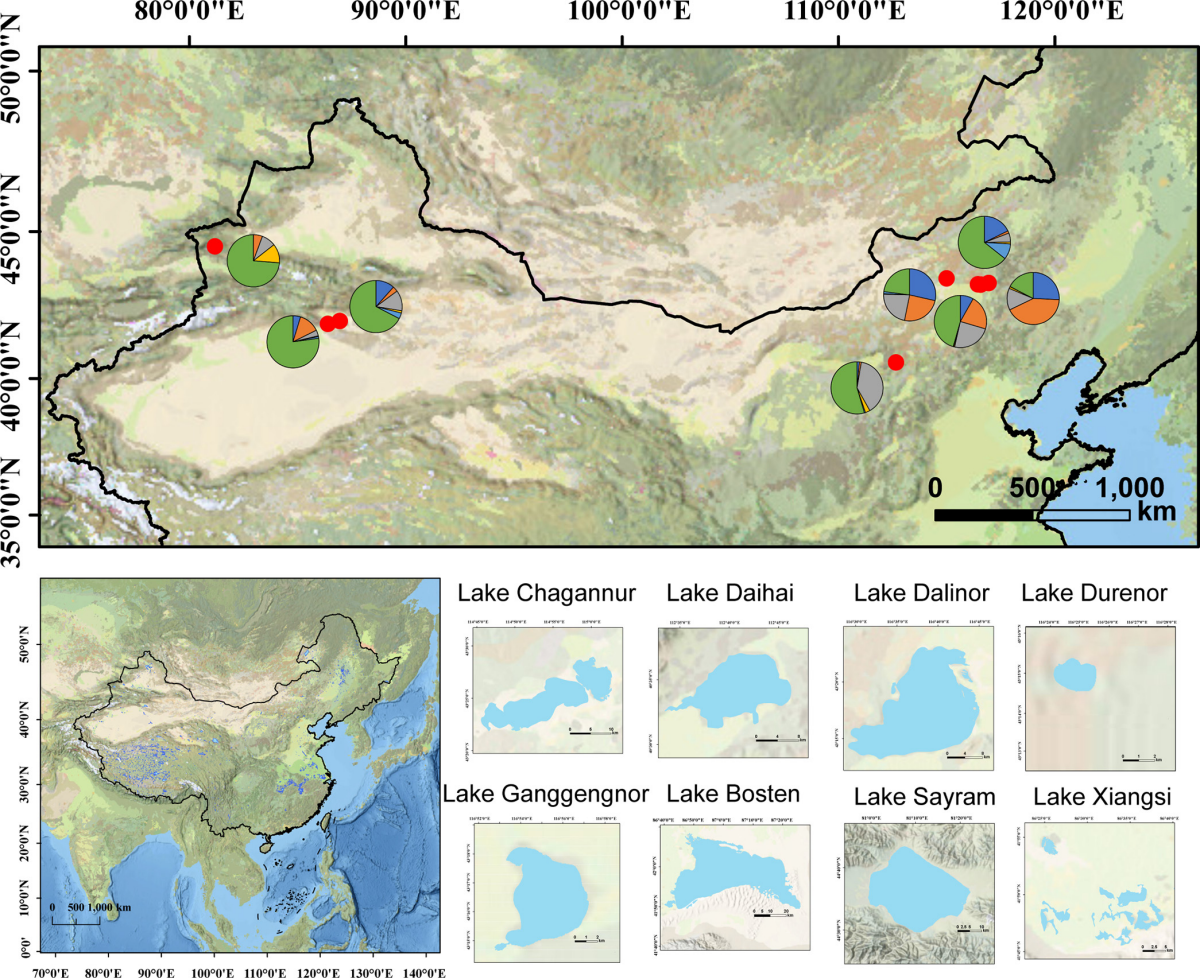
Sampling sites of the microeukaryotic community on the Inner Mongolia-Xinjiang Plateau. The pie charts present the relative abundance of consumers (orange), saprotrophs (yellow), parasites (blue), photosynthetic organisms (green), mixotrophs (wathet blue), and unclassified organisms (gray).

Sampling sites were located on the Inner Mongolia-Xinjiang Plateau (19.75°N to 47.58°N, 110.41°E to 126.92°E), which has a high diversity of lake types and experiences dramatic climate change (Fig. 8). In this study, surface water samples (*n* = 75) of lakes were collected from the Inner Mongolia region (September 2018) and the Xinjiang region (September 2019), including Lake Chagannur, Lake Daihai, Lake Dalinor, Lake Durenor, Lake Ganggengnor, Lake Bosten, Lake Sayram, and Lake Xiangsi (see Table S1 in the supplemental material). Physicochemical parameters, including temperature, pH, dissolved oxygen (DO), and salinity, were measured *in situ* at 0.5 m below the surface with a multiparameter water quality probe (YSI 6600; Yellow Spring Instruments, Yellow Springs, OH, USA).

The samples were transported immediately to the laboratory on ice in the dark for further analysis. The concentrations of total nitrogen (TN) were determined through colorimetry after digestion. Total phosphorus (TP) and ammonium (NH_4_^+^) were measured using a continuous flow analyzer (San ++ system; Skalar, Breda, the Netherlands) following the manufacturer’s instructions.

Approximately 300 mL of lake water was filtered through a 200-μm mesh to remove larger particles and macroplankton and then filtered through 0.22-μm polycarbonate filters (47 mm diameter; Millipore, Billerica, MA, USA) to collect microeukaryotes. Filters were stored at −80°C until further processing.

The mean monthly temperature (MMT), mean annual temperature (MAT), mean monthly precipitation (MMP), and mean annual precipitation (MAP) of the sampling site were extracted from Climatic Research Unit time series (CRU TS) version 4.05 of month-by-month variation (70). The monthly self-calibrating Palmer drought severity index (MPDSI) and annual self-calibrating Palmer drought severity index (APDSI) were introduced to assess the drought effect (71). PDSI values between −0.5 and 0.5 indicate near-normal conditions, values larger than 0.5 indicate wet conditions, and values lower than −0.5 indicate dry conditions (72). The mean monthly values were represented by value of sampling month, and the mean annual values were the average of 12 months before sampling date.

### DNA extraction and sequencing.

DNA was extracted using a FastDNA Spin kit for soil (MP Biomedicals, Solon, OH, USA) according to the manufacturer’s instructions. The 18S rRNA genes were amplified by PCR using the universal eukaryote primers Ek-NSF573 (5′-CGCGGTAATTCCAGCTCCA-3′) and Ek-NSR951 (5′-TTGGYRAATGCTTTCGC-3′) targeting the V4 region of most aquatic microeukaryote 18S rRNA genes (73). PCR amplification was performed using a touchdown program as described previously (55). The amplicons were then sent for sequencing on an Illumina HiSeq platform at Beijing Genomics Institute (Shenzhen, China). Sequences were deposited at the NCBI under BioProject accession numbers PRJNA755855 and PRJNA825691.

Raw fastq files were demultiplexed using the barcode sequence with the exact barcode matching parameter using div-utils (version 0.0.1; https://github.com/jameslz/div-utils/blob/master/div-utils). Quality filtering used Trimmomatic (version 0.36) with the following criteria: (i) bases off the start and end of a read below a threshold quality (score < 2) were removed; and (ii) the reads were truncated at any site receiving an average quality score of <15 over a 4-bp sliding window, discarding the truncated reads that were shorter than 36 bp. Paired reads were merged using USEARCH (version 11.2.64) (74) with the default parameters. Reads that could not be merged were discarded. The merged reads with more than 2 nucleotide mismatches in primer matching were removed. The primer sequences were removed from the merged reads.

Zero-radius operational units (ZOTUs) were generated using the USEARCH UNOISE algorithm, and the chimeric sequences were removed in the denoising procedure. The overall low-quality clean reads were first filtered using USEARCH fastq_filter with parameter fastq_maxee:1, the unique reads from the merged high overall quality reads were obtained and clustered using unoise3 subcommand with parameter -minsize 8, and a 97% similarity value was used to construct the ZOTU table by mapping the clean reads to representative sequences. Representative sequences of each ZOTU were aligned against the SILVA 132 database (BLAST threshold E value = E^−6^) for taxonomic annotation. ZOTUs identified as multicellular animals (Metazoa) and plants (Streptophyta) were removed. Based on the aligned taxonomy, the functional trophic groups were attributed to ZOTUs, which were divided into five trophic functional groups, including consumers, saprotrophs, parasites, photosynthetic organisms, and mixotrophs. The trophic division of microeukaryotes was based on the classification from Adl et al. and Keck et al. (75 and 76, respectively).

### Statistical analyses.

Alpha diversity indices were computed using the diversity function in the vegan R package after random resampling. The Kruskal-Wallis test was used to test the spatial differences in the environmental variables and ZOTU richness and Shannon diversity index by using the kruskal.test (stats R package) and kruskalmc (pgirmess R package) functions. The spatial and salinity effects of the microeukaryotic community were evaluated by permutational multivariate analysis of variance (PERMANOVA) using the adonis function in the vegan R package based on Bray-Curtis dissimilarity. Stepwise multiple-regression analysis was conducted to identify the impact factors of ZOTU richness and Shannon index using the lm and step functions in the stats R package. The microeukaryotic community composition was visualized using nonmetric multidimensional scaling (NMDS) based on Bray-Curtis dissimilarity. Variation partitioning analysis (VPA) was used to quantify the influence of physicochemical factors, climatic effects, and geographic patterns on microeukaryotic community variation. Canonical correlation analysis (CCA) was also used to test the correlation between environmental variables and microeukaryotic community structure. The differences in the abundances of microeukaryotes between freshwater and salt lakes were analyzed in the DESeq2 R package applied to raw count data (77). Changes in trophic groups are reported using the logarithmic (log_2_) fold change value, which expresses how much the number of reads changes between freshwater lakes (the reference) and salt lakes. The Wald test was used to test the significant difference in abundance, and all *P* values were adjusted for multiple-hypothesis testing using the method of Benjamini and Hochberg.

### Habitat niche breadth.

The niche breadth of microeukaryotic plankton communities was calculated using Levins’ niche breadth index (B) equation, $B_{j}=1/\overset{N}{\underset{i=1}{\sum }}P_{\mathrm{ij}}{}^{2}$ (78), where *B_j_* indicates the habitat niche breadth of ZOTU *j* in a metacommunity, *N* represents the total number of communities in each metacommunity, and P*_ij_* is the proportion of ZOTU *j* in community *i*. A ZOTU with a high B value indicates a wide habitat niche breadth. The community-level B value (*B*com) was calculated as the average of B values from all taxa occurring in one given community. The microeukaryotic community with a wide niche breadth is thought to be more metabolically flexible at the community level. The niche breadth index was conducted using the niche.width function from the spaa R package.

### Random forest.

To explore whether microeukaryotes can be used as biomarkers to indicate lake changes during climate change, we constructed RF models for salinity and APDSI using the high-throughput sequencing information of microeukaryotes. We constructed an RF model using the relative abundances of microeukaryotic taxa at different taxonomic resolutions (class, order, and ZOTU) as predictors and salinity and APDSI in lake ecosystems as response data by using the 10-fold cross-validation of the rfcv function in the randomForest package in R (ntree = 1,000) with five repeats (79). For each RF model, 70% of the data set was randomly separated into a training set, with the remaining 30% used as a testing set. Based on 10-fold cross-validation, the optimum feature set size was determined for different taxonomic resolutions to reconstruct the new model (80).

To further estimate the contributions of individual environmental variables to the microeukaryotic diversity and composition, we also regressed the environmental variables against the Shannon index and community composition using the randomForest package in R (ntree = 1,000). The community composition was represented by MDS1 of NMDS based on Bray-Curtis distance.

### Molecular ecological network construction.

To explore the network complexity and stability of the microeukaryotic community at different salinity gradients, the molecular ecological networks (MENs) of freshwater and salt lakes were constructed by the Molecular Ecological Network Analysis Pipeline (MENAP; http://ieg4.rccc.ou.edu/MENA/) (81, 82). All MENs were constructed on the basis of Pearson correlations of log-transformed ZOTU abundances, followed by a random matrix theory (RMT)-based approach that determines the correlation cutoff threshold in an automatic fashion. The MEN was constructed based on the ZOTU abundances in the 40 and 35 samples collected from freshwater lakes and salt lakes as the freshwater and salt networks, respectively. To ensure the reliability of the correlation calculation and reduce the complexity of the data sets, only the ZOTUs that occurred in at least 40% of the samples were included in the correlation calculation. The networks were visualized in Gephi version 0.9.2.

Further network topological properties were calculated in the MENAP to characterize the topological structure of the MENs, including *R*^2^ values of the power law fitting of node degrees, average degree, average clustering coefficient, average path distance, connectedness, modularity, etc. Meanwhile, 100 random networks were generated for each empirical network following the Maslov-Sneppen procedure (83) in the MENAP. The means and standard deviations of these properties from the 100 randomizations were calculated and compared with those from the corresponding empirical network. To explore the effect of salinity on network stability, the robustness of the network was calculated in the igraph R package (39). The more quickly robustness degraded when nodes were randomly removed, the lower the stability of the network.

To explore the role of individual nodes within the network, the within-module connectivity (Zi is the mean of nestedness) and among-module connectivity (Pi) (84) were calculated in the MENAP and used for classification of its topological roles in the network. The module hubs (Zi ≥ 2.5, Pi < 0.62), connectors (Zi < 2.5, Pi ≥ 0.62), and network hubs (Zi ≥ 2.5, Pi ≥ 0.62) were identified based on previous studies (85, 86). Those nodes are referred to as keystone nodes, and the other nodes were categorized as peripherals.

### Microeukaryotic community assembly processes.

To infer the ecological processes that regulate microeukaryotic community assembly processes, the β-nearest taxon index (βNTI) and Bray-Curtis-based Raup-Crick (RC_Bray_) were calculated by the null model (87, 88). βNTI was calculated using the comdistnt function from the picante R package (89), and RC_Bray_ was calculated by the determination of the deviation between the empirically observed Bray-Curtis data and the null distribution using the vegan package. When |βNTI| was >2, microeukaryotic community assembly was dominated by deterministic processes, including variable selection (βNTI > 2) and homogeneous selection (βNTI of less than −2). However, |βNTI| values below 2 indicated the dominance of stochastic processes, including homogenizing dispersal (RC_Bray_ of less than −0.95), dispersal limitation (RC_Bray_ > 0.95), and undominated processes (−0.95 < RC_Bray_ < 0.95) (30).

A partial Mantel test was used to estimate the effect of physicochemical, climatic, and geographic parameters on the βNTI after controlling for the other two effects. Partial Mantel tests were conducted using the mantel.partial function from the vegan R package.

### Structural equation modeling.

Structural equation modeling (SEM) was used to explore the direct and indirect relationships among physicochemical variables, climatic factors, and the microeukaryotic community. We constructed *a priori* models based on the known effects of physicochemical and climatic factors on microeukaryotic diversity and composition and tested whether the data fit these models using the lavaan package (90). The microeukaryotic diversity and composition were represented by the Shannon index and MDS1 of NMDS based on Bray-Curtis distance, respectively. To assess the goodness of fit, the χ^2^ test was used to evaluate the difference between the observed and estimated by model covariance matrices. The other parameters were also used to assess model fit, including the root mean square error of approximation (RMSEA), comparative fit index (CFI), and goodness-of-fit index (GFI). RMSEA of <0.08, CFI of >0.95, and GFI of >0.90 indicate a good fit of SEM.

### Data availability.

Raw Sequence data were deposited at the NCBI (https://www.ncbi.nlm.nih.gov/) under BioProject accession numbers PRJNA755855 and PRJNA825691.
