# Supplementary material for: Climate Change Causes Salinity To Become Determinant in Shaping the Microeukaryotic Spatial Distribution among the Lakes of the Inner Mongolia-Xinjiang Plateau
Source: Microbiol Spectr. 2023 Jun 12;11(4):e03178-22. doi: 10.1128/spectrum.03178-22 (PMC10434070; doi:10.1128/spectrum.03178-22)
Supplement: Supplemental file 1 — Supplemental material. Download spectrum.03178-22-s0001.pdf, PDF file, 1.2 MB [file spectrum.03178-22-s0001.pdf]

Supplementary information

Title: Climate change causes salinity to become determinant in shaping the microeukaryotic spatial distribution among the lakes of the Inner Mongolia-Xinjiang Plateau

Authors: Changqing Liu, Fan Wu, Xingyu Jiang, Yang Hu, Keqiang Shao, Xiangming Tang, Boqiang Qin, Guang Gao*

*Corresponding author: State Key Laboratory of Lake Science and Environment, Nanjing Institute of Geography and Limnology, Chinese Academy of Sciences, Nanjing 210008, China. Tel: +0086-25-86882187; Email: guanggao@niglas.ac.cn

Fig. S1 Physicochemical properties of the sampled lakes. CHA, DAI, DAL, DUR, GAN, BOS, SAY and XIA represent Lake Chagannur, Lake Daihai, Lake Dalinor, Lake Durenor, Lake Ganggengnor, Lake Bosten, Lake Sayram and Lake Xiangsi, respectively.

Fig. S2 Climatic parameters of the sampled lakes. CHA, DAI, DAL, DUR, GAN, BOS, SAY and XIA represent Lake Chagannur, Lake Daihai, Lake Dalinor, Lake Durenor, Lake Ganggengnor, Lake Bosten, Lake Sayram and Lake Xiangsi, respectively.

Fig. S3 Area changes in the sampled lakes from 1960 to 2020. (a) - (h) represent Lake Chagannur, Lake Daihai, Lake Dalinor, Lake Durenor, Lake Ganggengnor, Lake Bosten, Lake Sayram and Lake Xiangsi, respectively.

Fig. S4 The (a) OTU richness and (b) Shannon diversity index of microeukaryotic communities among the lakes of the Inner Mongolia-Xinjiang Plateau. CHA, DAI,

DAL, DUR, GAN, BOS, SAY and XIA represent Lake Chagannur, Lake Daihai, Lake Dalinor, Lake Durenor, Lake Ganggengnor, Lake Bosten, Lake Sayram and Lake Xiangsi, respectively.

Fig. S5 Relationship between OTU richness and latitude (a), longitude (b), altitude (c) and salinity (d).

Fig. S6 The results of variation partitioning models (VPAs) that were used to identify the effects of geographic patterns, climatic factors and physicochemical parameters.

Fig. S7 The relative importance of environmental variables to the microeukaryotic community (NMDS1) based on the RF model.

Fig. S8 Magnitude of change in taxonomic groups between the freshwater and salt lakes. Dark and gray bars represent groups for which the change was found significant ($P < 0.05$) and not significant ($P > 0.05$).

Fig. S9 Topological roles of nodes in the (a) freshwater and (b) salt networks based on Zi and Pi. Each symbol represents a node in one of the networks.

Fig. S10 The node degrees of different trophic types in freshwater and salt networks.

Fig. S11 The interspecies interactions among the different trophic groups in the (a) freshwater and (b) salt networks. Numbers outside and inside parentheses represent the proportion of edge numbers and negative edge numbers, and the red color represents the increase in the salt network.

Fig. S12 Relative importance of ecological processes that govern the assembly of microeukaryotic communities in freshwater and salt lakes.

Fig. S13 RF models were constructed using different taxonomic resolutions for lake salinity prediction by integrating microeukaryotic information. (a) Class, (b) order and (c) ZOTU numbers versus 10-fold cross-validation error. The dashed gray line marks the optimal cutoff for biomarker selection. The variable importance of the microeukaryotic biomarkers at the (d) class, (e) order and (f) ZOTU levels in RF models.

Fig. S14 The RF models were constructed using different taxonomic resolutions for APDSI prediction by integrating microeukaryotic information. (a) Class, (b) order and (c) ZOTU numbers versus 10-fold cross-validation error. The dashed gray line marks the optimal cutoff for biomarker selection. The variable importance of the microeukaryotic biomarkers at the (d) class, (e) order and (f) ZOTU levels in RF models.

Table S1 Characteristics of the lakes on the Inner Mongolia-Xinjiang Plateau.

Table S2 Environmental parameters in the lakes of the Inner Mongolia-Xinjiang Plateau.

Table S3 Taxonomic composition of microeukaryotic communities among the lakes of the Inner Mongolia-Xinjiang Plateau.

Table S4 Results of stepwise multiple regression models using OTU richness and Shannon index as response variables.

Table S5 Microeukaryotic community of variance explained by environmental variables according to canonical correlation analysis (CCA).

Table S6 Topological properties of the networks of salt and freshwater lakes.

Table S7 Partial Mantel test results showing the relationship between βNTI versus climatic and physicochemical and environmental distances for the microeukaryotic community after controlling for the other two explanatory distance constants.

Supplemental Figure

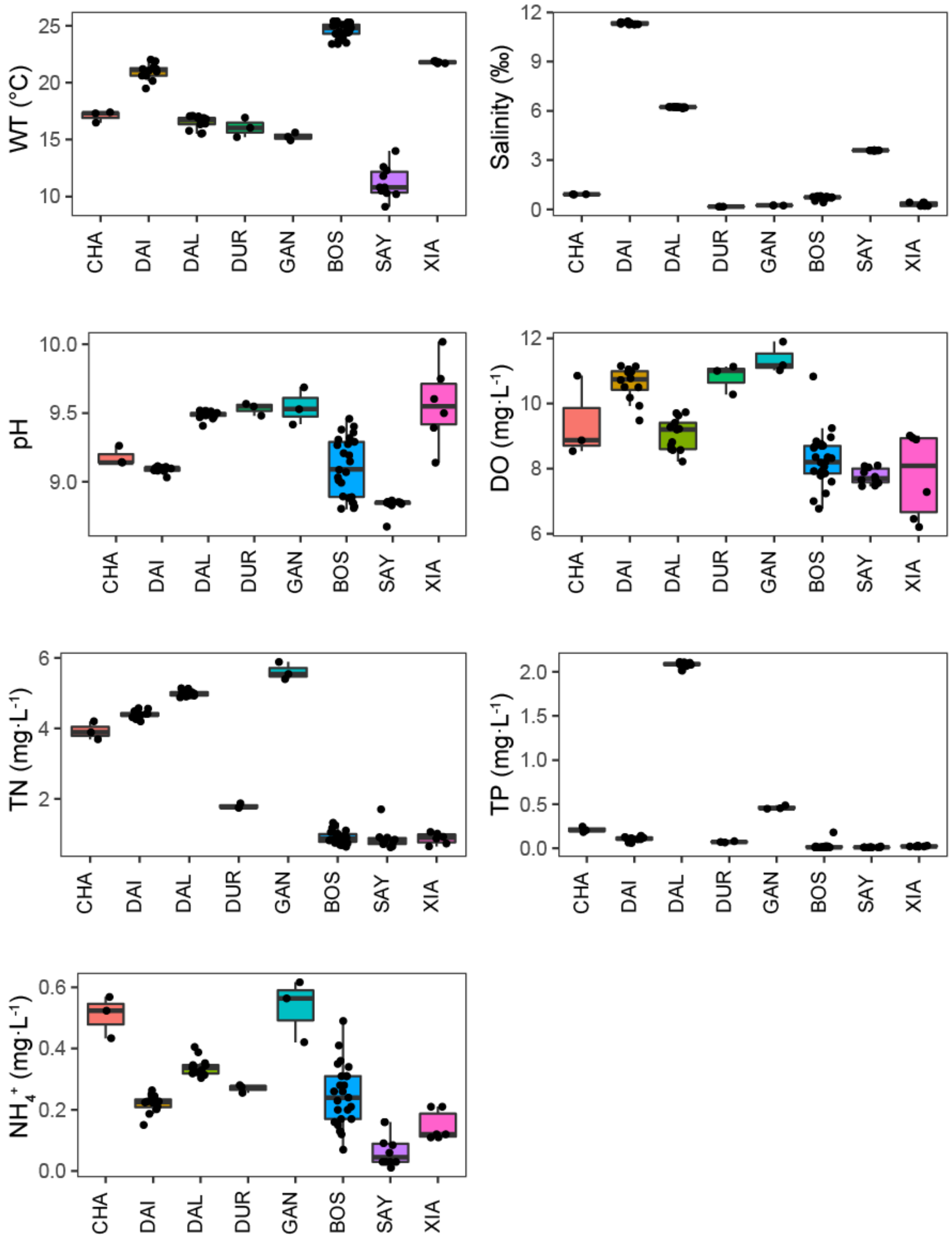

Fig. S1 Physicochemical properties of the sampled lakes. CHA, DAI, DAL, DUR, GAN, BOS, SAY and XIA represent Lake Chagannur, Lake Daihai, Lake Dalinor, Lake Durenor, Lake Ganggengnor, Lake Bosten, Lake Sayram and Lake Xiangsi, respectively.

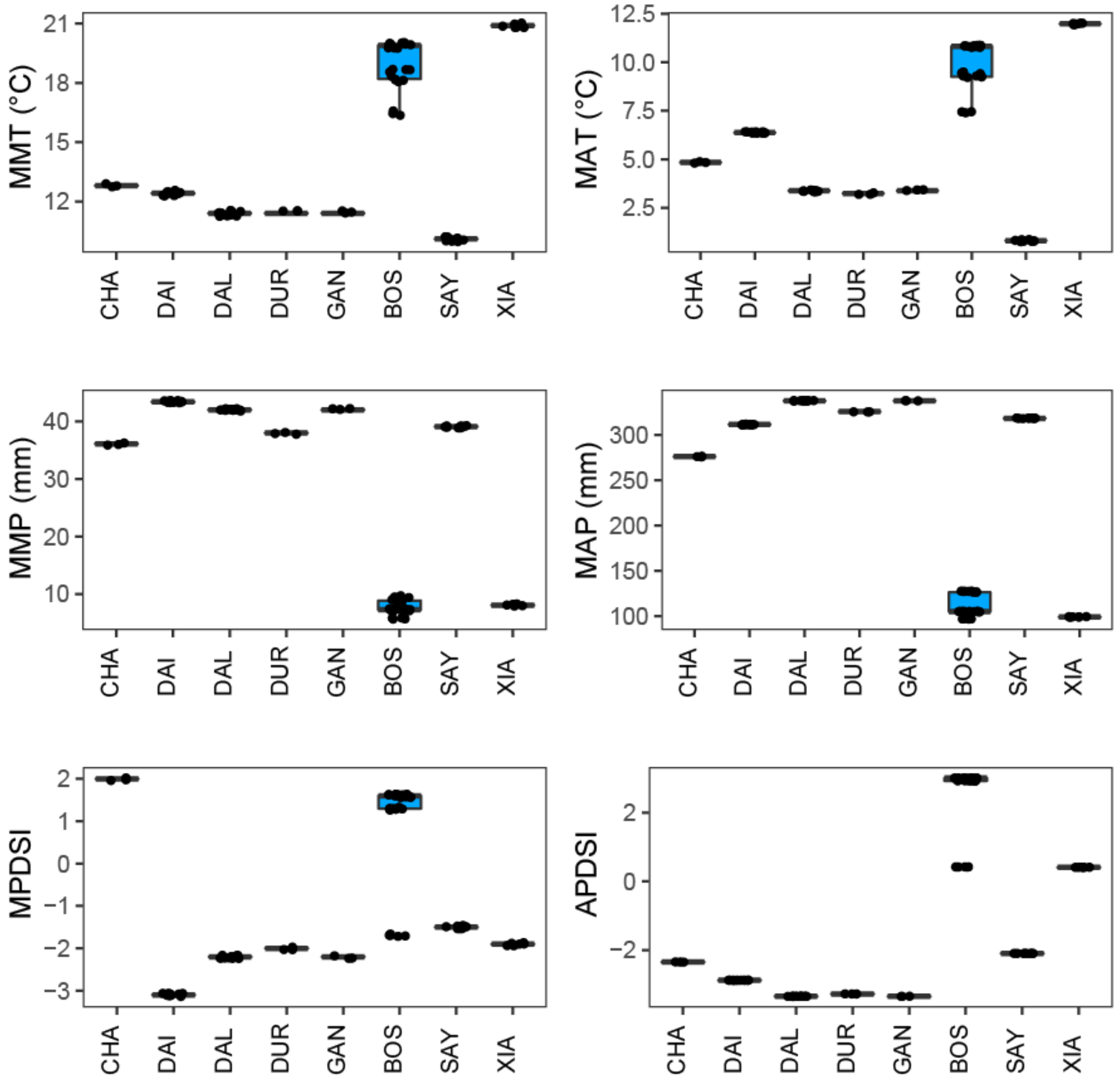

Fig. S2 Climatic parameters of the sampled lakes. CHA, DAI, DAL, DUR, GAN, BOS, SAY and XIA represent Lake Chagannur, Lake Daihai, Lake Dalinor, Lake Durenor, Lake Ganggengnor, Lake Bosten, Lake Sayram and Lake Xiangsi, respectively.

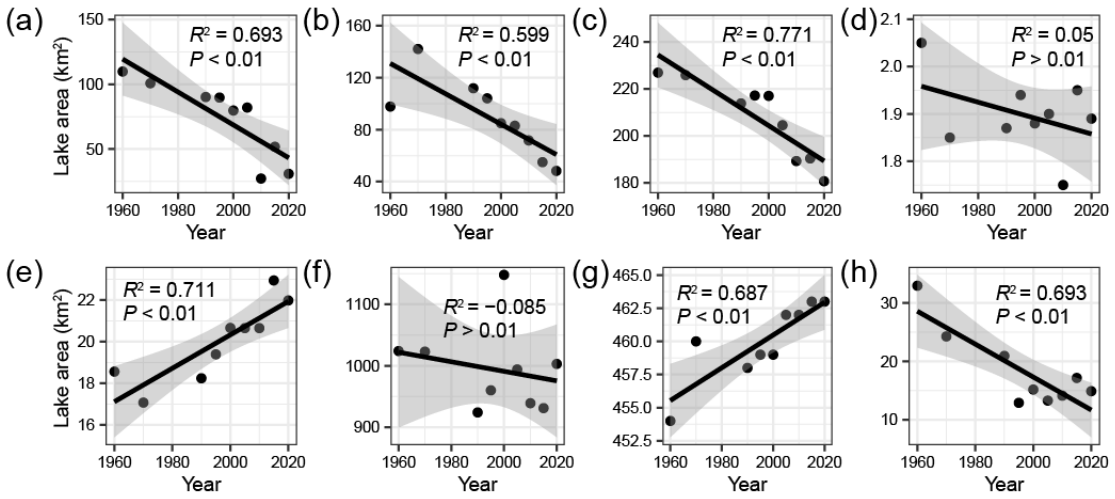

Fig. S3 Area changes in the sampled lakes from 1960 to 2020. (a) - (h) represent Lake Chagannur, Lake Daihai, Lake Dalinor, Lake Durenor, Lake Ganggengnor, Lake Bosten, Lake Sayram and Lake Xiangsi, respectively.

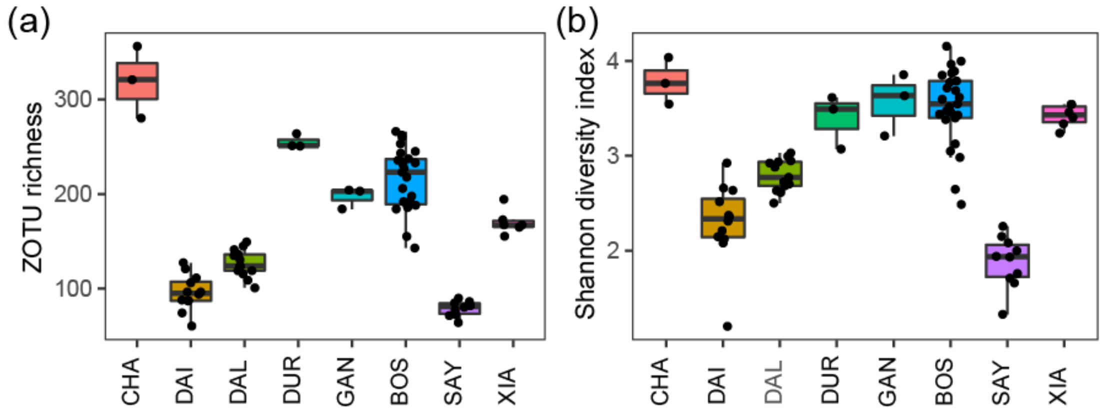

Fig. S4 The (a) ZOTU richness and (b) Shannon diversity index of microeukaryotic communities among the lakes of the Inner Mongolia-Xinjiang Plateau. CHA, DAI, DAL, DUR, GAN, BOS, SAY and XIA represent Lake Chagannur, Lake Daihai, Lake Dalinor, Lake Durenor, Lake Ganggengnor, Lake Bosten, Lake Sayram and Lake Xiangsi, respectively.

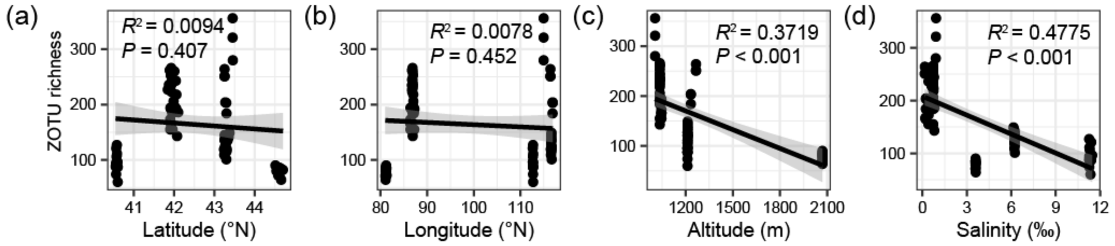

Fig. S5 Relationship between ZOTU richness and latitude (a), longitude (b), altitude (c) and salinity (d).

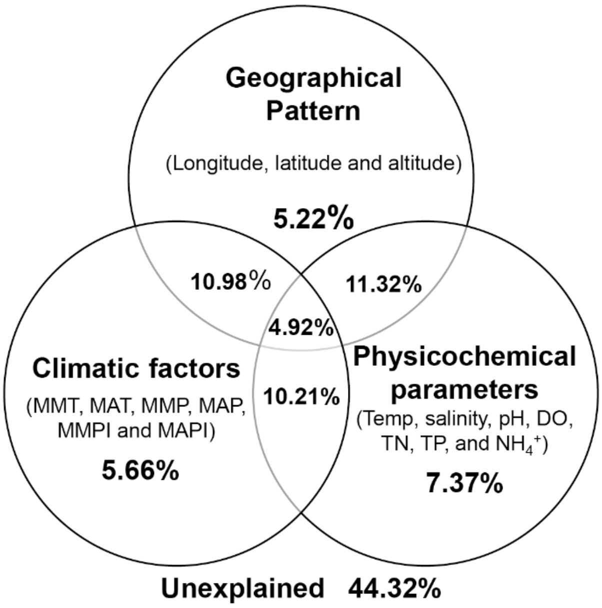

Fig. S6 The results of variation partitioning models (VPAs) that were used to identify the effects of geographic patterns, climatic factors and physicochemical parameters.

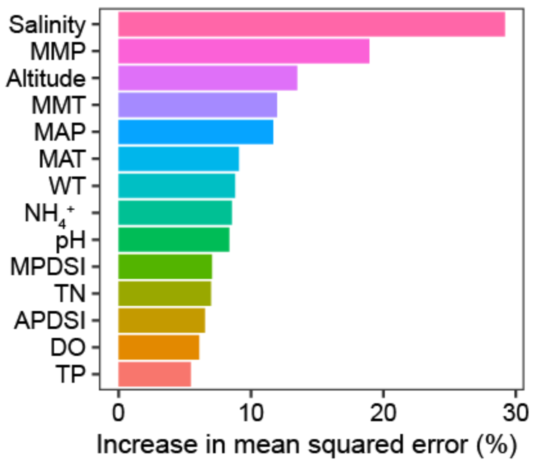

Fig. S7 The relative importance of environmental variables to the microeukaryotic community (NMDS1) based on the RF model.

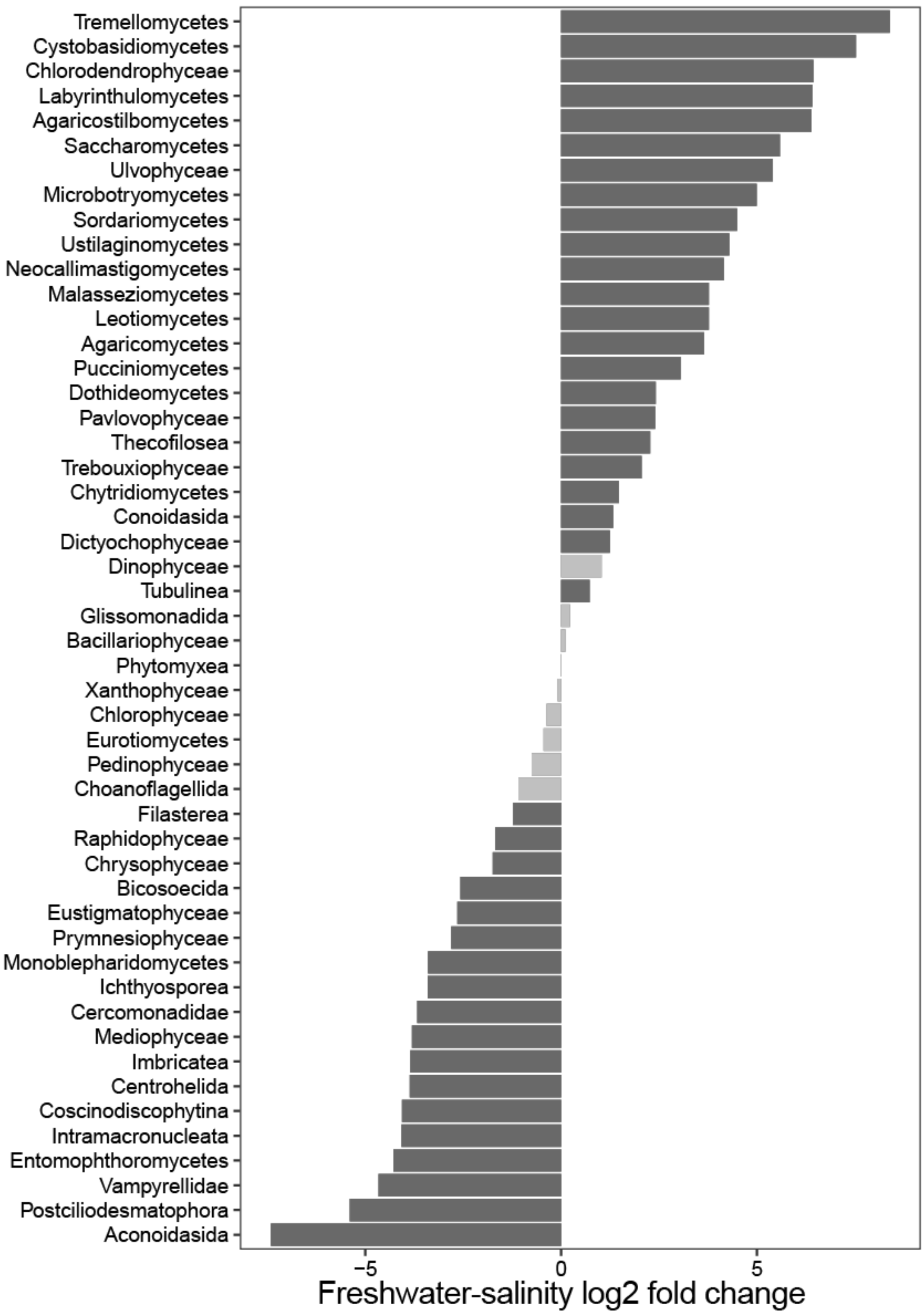

Fig. S8 Magnitude of change in taxonomic groups between the freshwater and salt lakes. Dark and gray bars represent groups for which the change was found significant ($P < 0.05$) and not significant ($P > 0.05$).

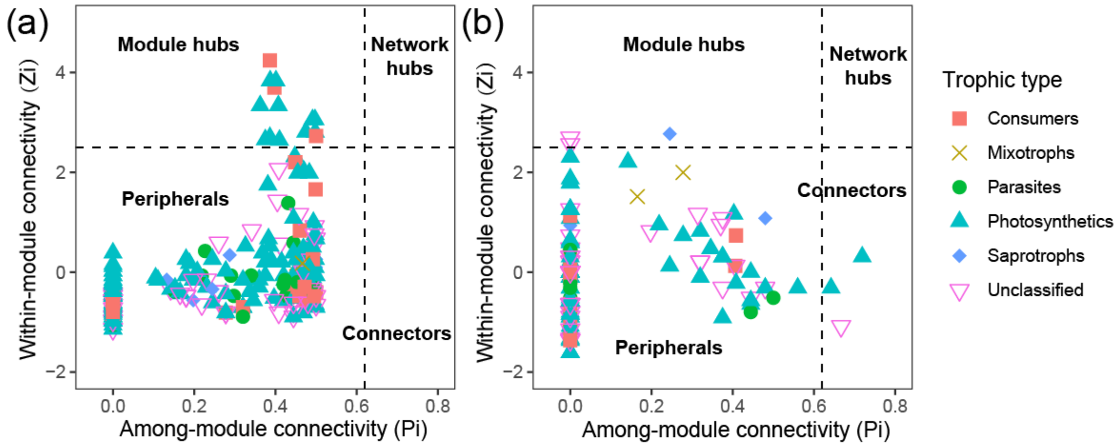

Fig. S9 Topological roles of nodes in the (a) freshwater and (b) salt networks based on Zi and Pi. Each symbol represents a node in one of the networks.

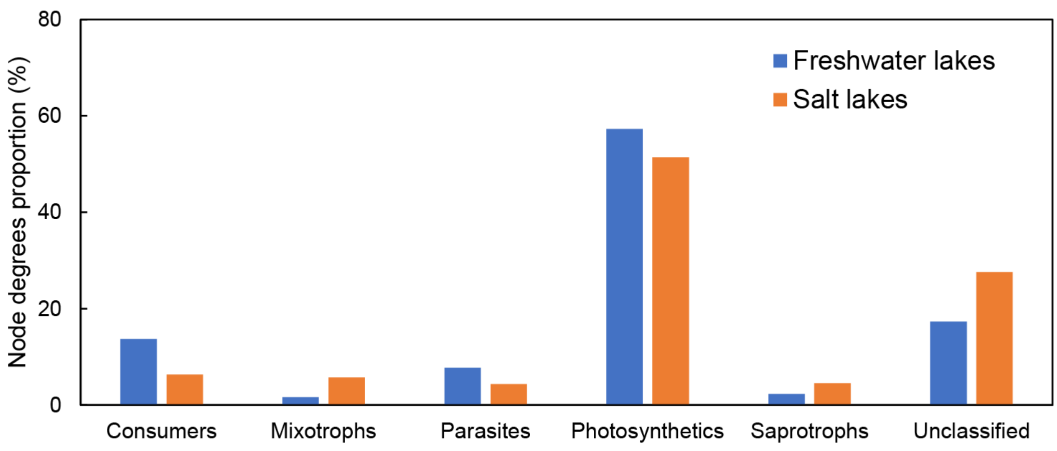

Fig. S10 The node degrees of different trophic types in freshwater and salt networks.

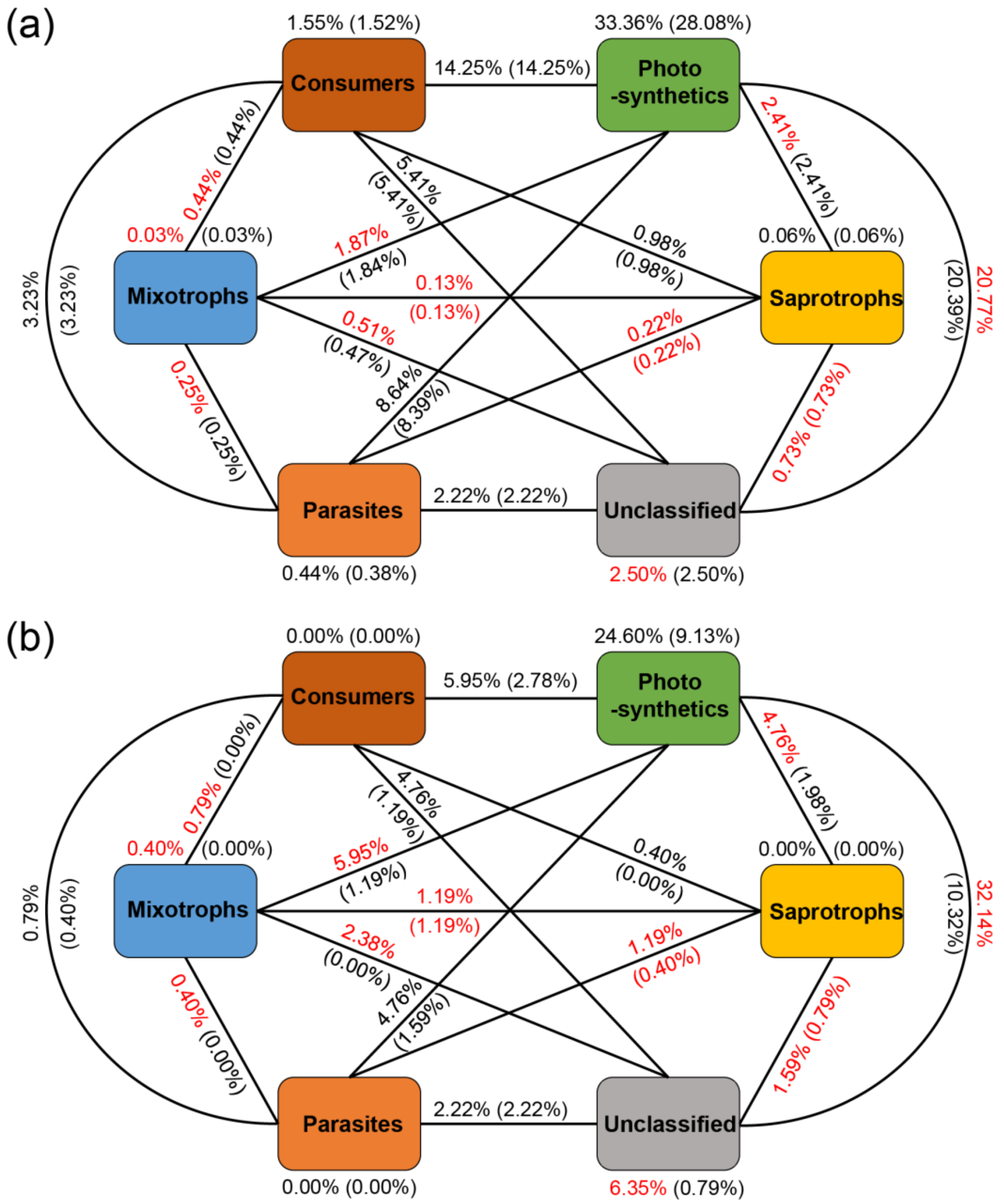

Fig. S11 The interspecies interactions among the different trophic groups in the (a) freshwater and (b) salt networks. Numbers outside and inside parentheses represent the proportion of edge numbers and negative edge numbers, and the red color represents the increase in the salt network.

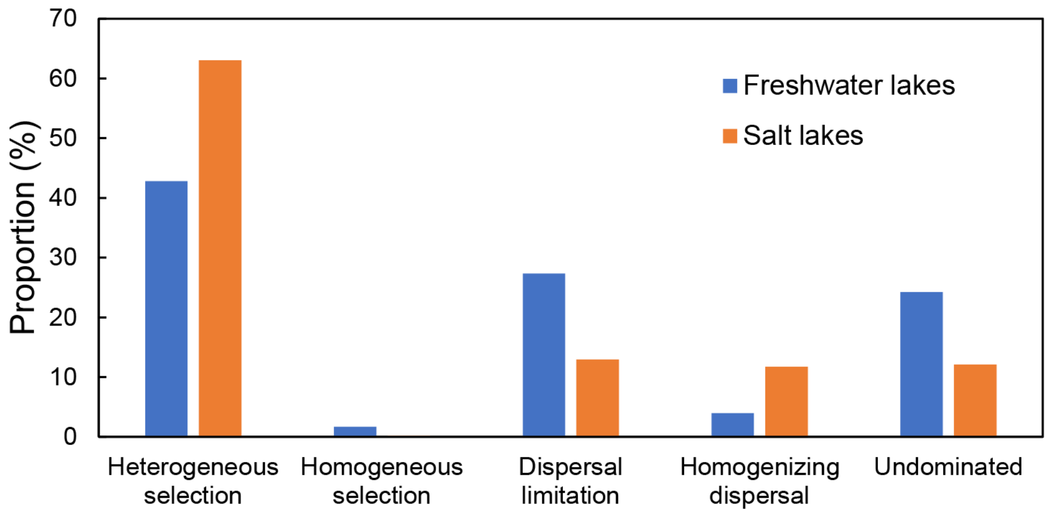

Fig. S12 Relative importance of ecological processes that govern the assembly of microeukaryotic communities in freshwater and salt lakes.

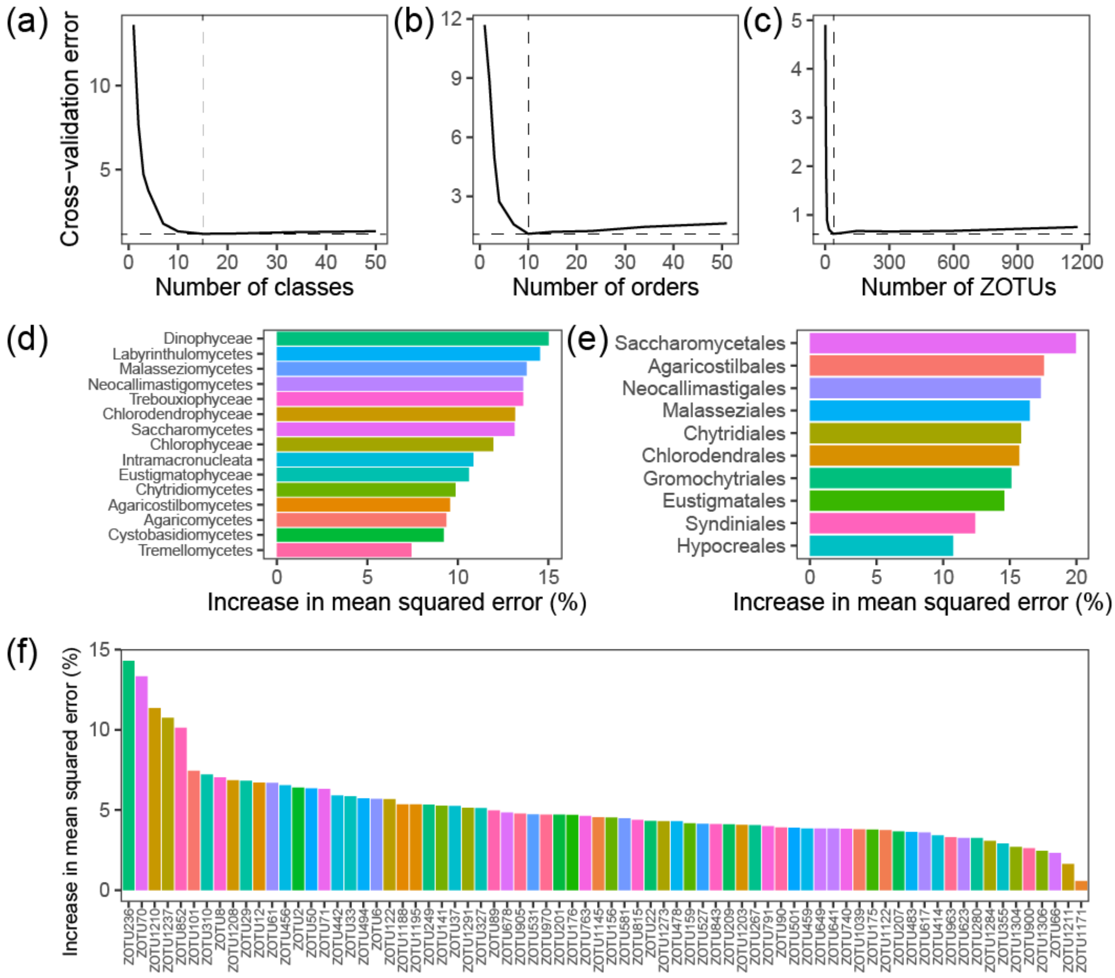

Fig. S13 RF models were constructed using different taxonomic resolutions for lake salinity prediction by integrating microeukaryotic information. (a) Class, (b) order and (c) ZOTU numbers versus 10-fold cross-validation error. The dashed gray line marks the optimal cutoff for biomarker selection. The variable importance of the microeukaryotic biomarkers at the (d) class, (e) order and (f) ZOTU levels in RF models.

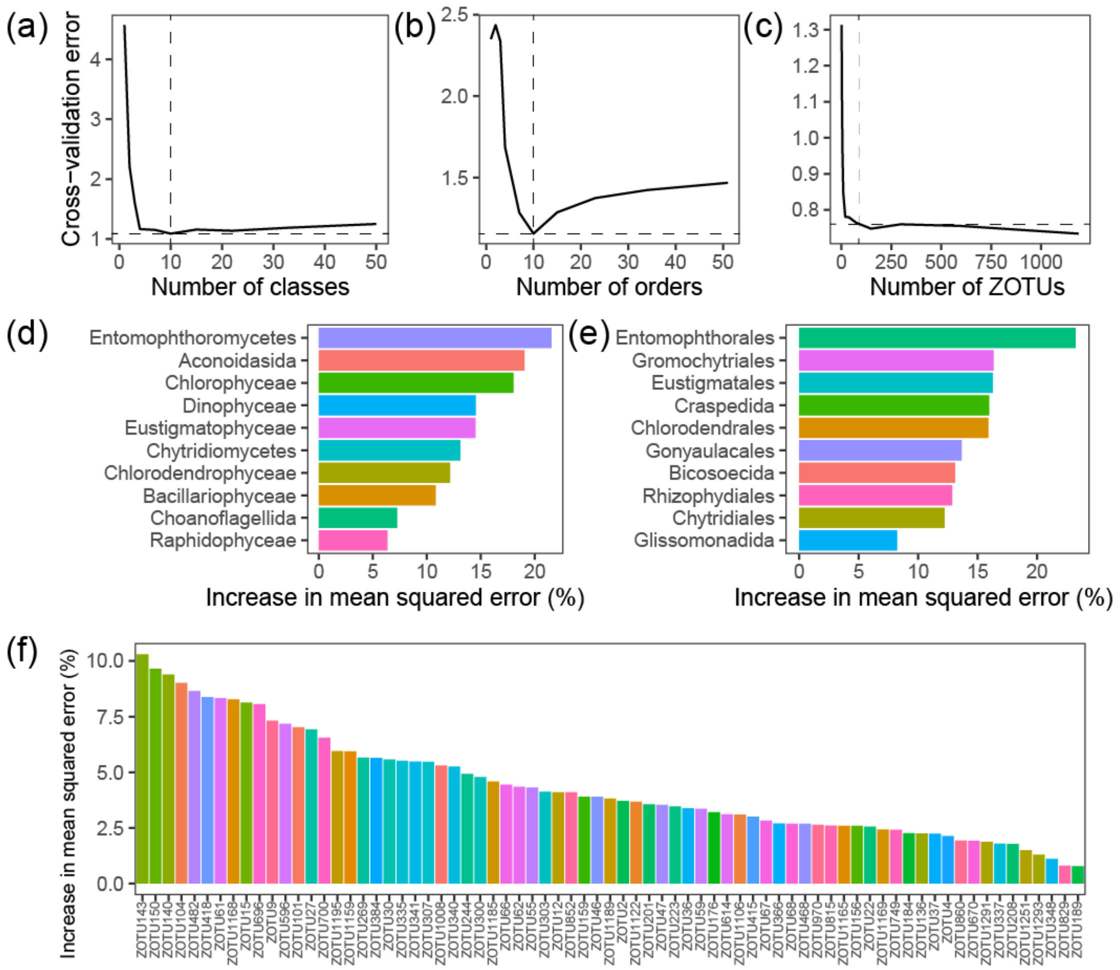

Fig. S14 The RF models were constructed using different taxonomic resolutions for APDSI prediction by integrating microeukaryotic information. (a) Class, (b) order and (c) ZOTU numbers versus 10-fold cross-validation error. The dashed gray line marks the optimal cutoff for biomarker selection. The variable importance of the microeukaryotic biomarkers at the (d) class, (e) order and (f) ZOTU levels in RF models.

Supplemental Table

Table S1 Characteristics of the lakes on the Inner Mongolia-Xinjiang Plateau.

Lake	Region	Sampling numbers[a]	Elevation (m)	Salinity Level	Water Surface Area (km^2)	Mean water depth (m)
Lake Chagannaoer	Inner Mongolia region	3	1013	freshwater	30	3.0
Lake Daihai	Inner Mongolia region	12	1221	salt	58	7.0
Lake Dalinuoer	Inner Mongolia region	13	1226	salt	190	7.5
Lake Durenaoer	Inner Mongolia region	3	1276	freshwater	2	3.5
Lake Ganggengnuoer	Inner Mongolia region	3	1243	freshwater	22	2.5
Lake Bosten	Xinjiang region	25	1036	freshwater	1003	9.5
Lake Sayram	Xinjiang region	10	2069	salt	463	46.4
Lake Xiangsi	Xinjiang region	6	1046	freshwater	14	2.5

a.The sampling number of each lake depended on the lake area and local condition.

Table S2 Environmental parameters in the lakes of the Inner Mongolia-Xinjiang Plateau.

Parameters type	Parameters	Mean	SD	Max	Min	Site[a]	Area[b]
Physicochemical parameters	Temp (℃)	19.60135	4.725795	25.4	9.1	P＜0.001	P＜0.001
	Salinity (‰)	3.6848	3.976831	11.46	0.16	P＜0.001	P＜0.001
	pH	9.2064	0.2845938	10.02	8.67	P＜0.001	P＜0.001
	DO ($mg·L^{-1}$)	8.944667	1.32643	11.9	6.21	P＜0.001	P＜0.001
	TN ($mg·L^{-1}$)	2.506349	1.905831	5.8836	0.62	P＜0.001	P＜0.001
	TP ($mg·L^{-1}$)	0.4169249	0.7721327	2.112	0.01	P＜0.001	P＜0.001
	$NH4^{+}$($mg·L^{-1}$)	0.2493853	0.1316934	0.6163	0.011	P＜0.001	P＜0.001
Climate parameters	MMT (℃)	14.72427	4.042603	20.9	10.1	P＜0.001	P＜0.001
	MAT (℃)	6.4244	3.708075	11.98	0.81	P＜0.001	P＜0.001
	MMP (mm)	27.288	16.60922	43.4	5.7	P＜0.001	P＜0.001
	MAP (mm)	233.2	106.2941	337.5	97.2	P＜0.001	P＜0.001
	MPDSI	-1.032	1.803162	2	-3.1	P＜0.001	P＜0.001
	APDSI	-0.8246667	2.601445	3.01	-3.35	P＜0.001	P＜0.001

a. The Kruskal–Wallis test was used to test the significant differences in the variables between the different lakes.

b. The Kruskal–Wallis test was used to test the significant difference in the variables between the Inner Mongolia and Xinjiang regions.

Table S3 Taxonomic composition of microeukaryotic communities among the lakes of the Inner Mongolia-Xinjiang Plateau.

Taxonomic affiliation	Reads	% of total reads	ZOTUs	% of total ZOTUs
**Alage**	**1703009**	**74.23%**	**460**	**38.72%**
Bacillariophyta	78164	3.41%	54	4.55%
Chlorophyta	1213852	52.91%	239	20.12%
Chrysophyceae	95034	4.14%	74	6.23%
Cryptophyceae	42077	1.83%	17	1.43%
Dinophyceae	244335	10.65%	47	3.96%
Eustigmatophyceae	24747	1.08%	14	1.18%
Other alage	4800	0.21%	15	1.26%
**Fungi**	**243596**	**10.62%**	**214**	**18.01%**
Ascomycota	14648	0.64%	20	1.68%
Basidiomycota	43674	1.90%	33	2.78%
Chytridiomycota	44163	1.93%	58	4.88%
Cryptomycota	125671	5.48%	60	5.05%
Other fungi	15440	0.67%	43	3.62%
**Protozoa**	**293586**	**12.80%**	**349**	**29.38%**
Cercozoa	96526	4.21%	117	9.85%
Ciliophora	82780	3.61%	108	9.09%
Protalveolata	88574	3.86%	39	3.28%
Other Protozoa	25706	1.12%	85	7.15%
**Unclassified**	**53910**	**2.35%**	**165**	**13.89%**
**Sum**	**2294101**	**100.00%**	**1188**	**100.00%**

Table S4 Results of stepwise multiple regression models using ZOTU richness and Shannon index as response variables.

Response variable	R^2 (%)	Predictor variable	F	P
**ZOTU richness**	**91.17**	Salinity	293.3331	**P < 0.001**
		WT	208.2925	**P < 0.001**
		MPDSI	82.0493	**P < 0.001**
		MAP	33.9639	**P < 0.001**
		MAT	26.5938	**P < 0.001**
		APDSI	9.1777	0.003513
		MMP	8.0465	0.006073
		DO	5.3652	0.023706
		MMT	4.1175	0.046542
**Shannon index**	**72.24**	Salinity	112.4123	**P < 0.001**
		MAT	45.0947	**P < 0.001**
		TP	20.351	**P < 0.001**
		MMP	4.2989	0.04182

Table S5 Microeukaryotic community of variance explained by environmental variables according to canonical correlation analysis (CCA).

Explained variance	Predictor variables	F	P
54.09%	Salinity	17.0975	＜0.001
	TP	15.2902	＜0.001
	WT	15.202	＜0.001
	TN	12.8846	＜0.001
	MPDSI	6.3378	＜0.001
	MAT	5.2459	＜0.001
	DO	3.1961	＜0.001
	pH	2.5192	＜0.001

Table S6 Topological properties of the networks of salt and freshwater lakes.

Network Indices	Freshwater network		Salt network	
	Empirical network	Random Networks	Empirical network	Random Networks
Similarity threshold	0.81	/	0.81	/
Total nodes	252	/	134	/
Total links	3159	/	252	/
R square of power law	0.637	/	0.803	/
Average connectivity	25.071	/	3.761	/
Average clustering coefficient	0.137	0.479 ± 0.009	0.252	0.035 ± 0.010
Average path distance	2.185	2.038 ± 0.007	4.369	3.630 ± 0.067
Harmonic geodesic distance	1.981	1.897 ± 0.004	3.413	3.156 ± 0.044
Geodesic efficiency	0.505	0.527 ± 0.001	0.293	0.317 ± 0.004
Transitivity	0.098	0.274 ± 0.003	0.329	0.050 ± 0.010
Connectance	1	1.000 ± 0.000	0.618	0.943 ± 0.035
Modularity	0.18	0.120 ± 0.004	0.67	0.485 ± 0.010
Positive links	192	/	171	/
Negative links	2967	/	81	/
Proportion (Positive/total)	0.061	/	0.679	/

Table S7 Partial Mantel test results showing the relationship between βNTI versus climatic and physicochemical and environmental distances for the microeukaryotic community after controlling for the other two explanatory distance constants.

Variable(s)	Variance
**Control climatic + geographic pattern effect**	
physicochemical effect	19.84%*
**Control physicochemical + geographic pattern effect**	
climatic effect	-19.69%
**Control physicochemical + climatic effect**	
geographic pattern effect	21.09%**

*, ** and *** indicate significant differences at $P < 0.05$, $P < 0.01$ and $P < 0.001$, respectively.
